# In Celiac Disease, a Subset of Autoantibodies against Transglutaminase Binds Toll-Like Receptor 4 and Induces Activation of Monocytes

**DOI:** 10.1371/journal.pmed.0030358

**Published:** 2006-09-19

**Authors:** Giovanna Zanoni, Riccardo Navone, Claudio Lunardi, Giuseppe Tridente, Caterina Bason, Simona Sivori, Ruggero Beri, Marzia Dolcino, Enrico Valletta, Roberto Corrocher, Antonio Puccetti

**Affiliations:** 1 Section of Immunology, Department of Pathology, University of Verona, Verona, Italy; 2 Section of Internal Medicine, Department of Clinical and Experimental Medicine, University of Verona, Verona, Italy; 3 Section of Histology, Department of Experimental Medicine, University of Genova, Genova, Italy; 4 Immunology Unit, Institute G. Gaslini, Genova, Italy; 5 Department of Pediatrics, University Hospital of Verona, Verona, Italy; Institute for Clinical Molecular Biology, Germany

## Abstract

**Background:**

Celiac disease is a small intestine inflammatory disorder with multiple organ involvement, sustained by an inappropriate immune response to dietary gluten. Anti-transglutaminase antibodies are a typical serological marker in patients with active disease, and may disappear during a gluten-free diet treatment. Involvement of infectious agents and innate immunity has been suggested but never proven. Molecular mimicry is one of the mechanisms that links infection and autoimmunity.

**Methods and Findings:**

In our attempt to clarify the pathogenesis of celiac disease, we screened a random peptide library with pooled sera of patients affected by active disease after a pre-screening with the sera of the same patients on a gluten-free diet. We identified a peptide recognized by serum immunoglobulins of patients with active disease, but not by those of patients on a gluten-free diet. This peptide shares homology with the rotavirus major neutralizing protein VP-7 and with the self-antigens tissue transglutaminase, human heat shock protein 60, desmoglein 1, and Toll-like receptor 4. We show that antibodies against the peptide affinity-purified from the sera of patients with active disease recognize the viral product and self-antigens in ELISA and Western blot. These antibodies were able to induce increased epithelial cell permeability evaluated by transepithelial flux of [^3^H] mannitol in the T84 human intestinal epithelial cell line. Finally, the purified antibodies induced monocyte activation upon binding Toll-like receptor 4, evaluated both by surface expression of activation markers and by production of pro-inflammatory cytokines.

**Conclusions:**

Our findings show that in active celiac disease, a subset of anti-transglutaminase IgA antibodies recognize the viral protein VP-7, suggesting a possible involvement of rotavirus infection in the pathogenesis of the disease, through a mechanism of molecular mimicry. Moreover, such antibodies recognize self-antigens and are functionally active, able to increase intestinal permeability and induce monocyte activation. We therefore provide evidence for the involvement of innate immunity in the pathogenesis of celiac disease through a previously unknown mechanism of engagement of Toll-like receptor 4.

## Abstract 2

A subset of anti-transglutaminase IgA antibodies recognize the viral protein VP-7, suggesting a possible involvement of rotavirus infection in the pathogenesis of celiac disease through a mechanism of molecular mimicry.

## Editors' Summary

**Background.:**

Celiac disease is an autoimmune, digestive disorder in which the small intestine (the part of the gut that absorbs nutrients from food) is damaged. In autoimmune diseases, the immune system, which normally provides protection against foreign invaders, attacks a person's own tissues. In celiac disease, this attack is triggered by eating food containing gluten, a mixture of proteins found in wheat, barley, and rye. To avoid malnutrition, people with celiac disease—about one in 100 people of north European descent—must follow a strict, lifelong gluten-free diet, one that avoids baked products, wheat, pasta, and many other foods. If they fail to do this, their immune system may attack not only their gut but also their brain, skin, joints, and other tissues, in part through the production of antibodies (autoantibodies) that recognize a protein (self-antigen) called tissue transglutaminase. Celiac disease is diagnosed also by looking for these autoantibodies in patients' blood when they are on a gluten-containing diet; they rapidly disappear when a gluten-free diet is adopted.

**Why Was This Study Done?:**

A gluten-free diet keeps celiac disease in check but does not cure it and is very difficult to follow. Even the minute amounts of gluten found in medicines, for example, can trigger the production of autoantibodies and active disease. But developing a cure is impossible without a better understanding of how celiac disease develops. Why, for example, do celiac disease patients make anti-transglutaminase antibodies? Were they made initially to ward off an infectious agent but unfortunately also recognized transglutaminase? In this study, the researchers asked whether “molecular mimicry”—cross-reactivity between self-molecules and foreign molecules on bacteria or viruses (pathogens)—might initiate celiac disease. They also asked whether innate immunity (the part of the immune system that responds quickly to general features on pathogens) as well as adaptive immunity (the production of antibodies and immune cells that recognize specific features on pathogens) is involved in the development of celiac disease.

**What Did the Researchers Do and Find?:**

The researchers purified antibodies from blood provided by patients with celiac disease when they were eating food containing gluten and when they were on a gluten-free diet. They used these to identify celiac peptide, a synthetic protein fragment that was recognized only by the antibodies made by patients with active disease. By searching a database of pathogen proteins, the researchers discovered that rotavirus protein VP-7 contains a very similar peptide; a search of a database of human proteins indicated that celiac peptide also resembles peptides found in tissue transglutaminase, Toll-like receptor 4 (TLR4; a protein involved in the innate immune response), and several other self-antigens. Patient antibodies purified through their ability to bind to celiac peptide also bound to VP-7 and to these self-antigens, and only patients with active disease made these antibodies. The researchers also investigated whether these anti-celiac peptide antibodies might affect the gut or the innate immune system. The antibodies increased the permeability of a layer of gut cells growing in a laboratory dish by interacting with the self-antigen desmoglein 1. This protein helps to make impermeable seals between the cells that line the gut so that food antigens in the gut cannot seep out into the tissues where the immune system might detect them. In addition, by binding to TLR4, the anti-celiac peptide antibodies activated monocytes—cells that function in both the innate and adaptive immune response.

**What Do These Findings Mean?:**

The finding that some anti-transglutaminase antibodies recognize the viral protein VP-7 could mean that rotavirus infection, which causes gastroenteritis, helps to initiate celiac disease in susceptible individuals through molecular mimicry. Furthermore, the identification of other self-antigens that contain peptides recognized by the antibodies made during active disease starts to explain why damage occurs outside the gut in people with celiac disease. The ability of these antibodies to recognize all these peptides could be coincidental, but the observation that the antibodies have relevant functional effects—the ability to increase intestinal permeability and to activate monocytes—makes this less likely. More research is needed to reveal exactly how infections and the innate immune response affect the development of celiac disease, but every piece of new information brings the possibility of a cure a little closer.

**Additional Information.:**

Please access these Web sites via the online version of this summary at http://dx.doi.org/10.1371/journal.pmed.0030358.

- US National Institute of Diabetes and Digestive and Kidney Diseases, information for patients on celiac disease
- MedlinePlus encyclopedia entries on celiac disease and on  autoimmunity
- Wikipedia pages on celiac disease and on autoimmunity (note that Wikipedia is a free online encyclopedia that anyone can edit)

## Introduction

Gluten enteropathy is a chronic systemic autoimmune disorder [1,2] sustained by an inappropriate immune response to dietary gluten [3], that may affect as many as 1%–3% of the European and North American population [4]. Genetically susceptible individuals develop autoimmune injury to the gut, skin, joints, liver, brain, heart, uterus, and other organs. In addition, celiac patients show an increased prevalence of other autoimmune diseases. Celiac disease (CD) is considered a model for autoimmune disorders because the crucial genetic and environmental factors responsible for its pathogenesis have been identified. It is well known that the interplay of four components induces enteropathy: gluten/gliadin, gluten-specific T cells, the major histocompatibility complex locus HLA-DQ, and the endogenous enzyme tissue transglutaminase (tTG) [1]. tTG can deaminate glutamine to glutamic acid, producing the negatively charged residues necessary for efficient binding to DQ2 and for T cell activation. Genetic, molecular, and functional studies have clarified the powerful Th1-dominated pro-inflammatory response that characterises the small intestine of patients with active CD. Moreover, patients with active CD have immunoglobulin (Ig)A and IgG antibodies directed against tTG [5]. The disease enters complete remission, and anti-tTG antibodies are no longer detectable when gluten is eliminated from the diet (gluten-free diet [GFD]) [6].

In addition to the activation of adaptive immune response, recent evidence suggests that innate immunity might play a role in the initial phases of CD [7]. Indeed, some gluten peptides seem to be able to directly activate the innate immune system [8]. Moreover, infectious agents are thought to play a role in the pathogenesis of the disease [9,10].

A key feature of the early stages of CD is the presence of increased intestinal epithelial cell permeability [1,10,11]. One of the mechanisms involved is related to tight-junction derangement [12]. However, alterations involving other types of junctional adhesion may contribute to the increased epithelial permeability.

Despite the huge progress in understanding the pathogenesis of CD, facilitated by the knowledge of the genetic background and of the well-defined environmental trigger gluten/gliadin, there are still many unknowns, including the presence of autoaggression in different tissues and the role played by infections and by innate immunity.

In this study, we have identified an autoantigen peptide that is recognized by serum Igs of patients with active disease (on a gluten-containing diet [GCD]), but not by patients on GFD. We show that antibodies against the peptide purified from the sera of patients recognize the rotavirus major neutralizing protein VP-7 [13] and self-antigens including tTG [14], human heat shock protein 60 (HSP60) [15], desmoglein 1 [16], and Toll-like receptor 4 (TLR4) [17,18]. The anti-peptide antibodies are pathogenetically relevant because of their ability to alter the intestinal barrier integrity and to activate monocytes through a previously unknown mechanism of TLR4 stimulation.

## Methods

### Patients

Sixty patients affected by CD were enrolled in the study. The first group included 22 patients with active disease, four males and 18 females, aged 1–38 (mean = 16) y, at diagnosis of biopsy confirmed CD and after a period of 5 mo to 9 y (mean = 5.6 y) of GFD. A second group of 38 CD patients, 12 males and 26 females, aged 1–56 (mean = 14) y, with active disease, was also studied. All the patients had serum anti-gliadin and anti-tTG antibodies. Antibodies against IgA, IgM, and IgG were not detected in the sera of all the patients. The duodenal histological damage at diagnosis ranged from grade 3a to 3c, according to Marsh's classification [19]. Patients on GFD were negative for anti-tTG IgA antibodies. The majority of patients suffered from gastrointestinal symptoms; the symptoms present at diagnosis were: gastrointestinal (diarrhoea, abdomen distension, and cramps) in 39 out of 60 patients, and/or extra-intestinal (iron deficiency anemia, dermatitis herpetiformis, spontaneous abortion, weight loss, astenia, growth disturbances, and myopathy) in 25 out of 60 patients. One of the patients with gastrointestinal symptoms had thrombocytopenia and no evidence of other autoimmune diseases, and another one suffered from demyelinating CNS disease [20,21]: in this latter patient, multiple sclerosis was excluded on the basis of clinical follow-up, cerebrospinal liquid analysis, and pattern of CNS demyelinization as seen at magnetic resonance imaging. Some patients presented only mild or minimal symptoms, and four patients were asymptomatic (silent CD) with familial history of CD. The patients with CD included in the study showed no evidence of co-existing autoimmune diseases. Human leukocyte antigen (HLA) haplotype was performed in 20 patients: 14 patients (70%) were HLA DQ2 positive, four patients (20%) were HLA DQ8 positive, and two patients (10%) were HLA DQ2/DQ8 positive. Control sera were obtained from 60 healthy age- and sex-matched individuals who had no evidence of autoimmunity, were negative for anti-tTG and anti-gliadin antibodies, and had no evidence of intestinal disease as assessed by biopsy.

Sera from 90 patients with systemic sclerosis, from 50 patients with systemic lupus erythematosus, and from 40 patients with Crohn disease were also used as controls for some experiments.

### Cell Culture

T84 cells obtained from the American Type Culture Collection (Manassas, Virginia, United States) were grown in a 5% CO_2_ humidified incubator at 37 °C on 162-cm^2^ flasks (Corning Costar, Acton, Massachusetts, United States) with media containing a 1:1 mixture of Ham's F-12 nutrient mixture and DMEM supplemented with 6% heat-inactivated fetal bovine serum, 15 mM HEPES, 14.3 mM NaHCO_3_, and antibiotics (penicillin and streptomycin)–antimycotics (pH 7.4). Cells were passaged weekly on reaching confluence. For experiments, cells were plated onto collagen-coated permeable supports, where they were fed every 3 d and used from days 7–14 (within fifth passage).

The 293T cells transfected with *TLR4* or *TLR9* as well as their culture medium were purchased from Cayla-Invivogen (Ruschlikon, Switzerland). Control untransfected 293T cells were from Cayla-Invivogen. The human cell line K562 was cultured under standard conditions (RPMI 10% FCS) [22].

### Library

The FliTrx random dodecamer peptide library [23], which displays peptides on the surface of Escherichia coli using the major bacterial flagellar protein (FliC) and thioredoxin (Lu), was purchased from Invitrogen (Carlsbad, California, United States) and screened with pooled Igs affinity purified from the sera of the 22 patients with active disease on GCD according to manufacturer's instructions (FliTrx Panning Kit, Invitrogen). After five sequential rounds of biopanning experiments, each preceded by a pre-panning step with pooled Igs obtained from the same 22 patients on GFD to remove bacteria that bound antibodies present in sera of patients with inactive disease, the enriched library was grown and single colonies picked, expanded, and induced with tryptophan to express the fusion peptides. Bacteria were then lysed in sample buffer and tested in Western blot with the pooled Ig fraction from patients on GCD to check for positive clones**.** DNA was extracted from positive clones and sequenced with an automated sequencing apparatus (Perkin Elmer, Norwalk, Connecticut, United States).

A set of 15 peptides, out of the 27 peptides obtained from the last biopanning round, was synthesized, excluding those peptides that shared a high degree of homology with each other (greater than 90%, which corresponds to ten out of 12 amino acid identities or conservative substitutions). The 15 synthetic peptides were used in an enzyme-linked immunosorbent assay (ELISA) to test individual patients' sera as well as control Igs. At the end of the screening procedure, we calculated the frequency of antibodies against the individual peptides. In most cases, the reactivity was present in approximately 5% of the patients; therefore, we did not consider these sequences anymore. In one case, reactivity against the synthetic peptide was present in approximately 65% of the patients' sera (celiac peptide). Since no other peptide was recognized by serum Igs at such frequency, we focused our attention on this particular peptide. The celiac peptide was already sequenced after the second biopanning round and three times after the fifth round, indicating that this reactivity is important since it was still present after five steps of enrichment of the peptide library. Moreover, the fact that we could obtain the same sequence from three independently picked clones suggests that this peptide is over-represented among the group of antibody-selected peptide epitopes. Similar behaviour has been observed in other screening procedures performed in our laboratory using the sera of patients with autoimmune diseases [23–26].

### Peptide Synthesis

All the synthetic peptides, including the celiac peptide, the rotavirus VP-7 peptide (VIQVGGSNVLDI), the TLR4 peptide (VLKMAGNSFQEN), the desmoglein 1-peptide (LSSLGGTASIGH), the tTG peptide (RIRVGQSMNMGS) and the irrelevant control peptide (VTLPKDSDVELP), were manually synthesized using the standard method of solid-phase peptide synthesis, which follows the 9-fluorenylmethoxycarbonyl (FMOC) strategy with minor modifications [27]. All the synthesized compounds were purified by reverse-phase high-performance liquid chromatography (RP-HPLC) and the molecular weights finally confirmed by electrospray mass spectrometry.

### Affinity Purification of Total Human Serum Immunoglobulins

Serum samples (1 ml per 3 ml of resin) were applied to T-Gel Adsorbent Columns (Pierce Biotechnology, Rockford, Illinois, United States). The columns were washed with binding buffer. Bound Igs were eluted with eluting buffer and dialyzed against PBS. All the reagents used for the purification procedure were supplied by Pierce (T Gel Purification Kit for total Ig purification). The purity of the preparations was assessed by SDS-PAGE followed by silver staining; sometimes, when albumin contamination was present in the purified preparation, an additional step was added using an Albumin Removal Kit (Pierce).The same procedure was used to purify rabbit Igs from rabbit antisera.

### Affinity Purification of Anti-Peptide Antibodies

The synthetic peptides (5 mg peptide per gram of dried Sepharose powder) were coupled to Sepharose 4B (Pharmacia, Uppsala, Sweden) according to the manufacturer's instructions. Serum Igs (see previous section) diluted in PBS were applied to the columns. The columns were washed with PBS. Bound Ig were eluted with 0.1 M glycine (pH 2.5) and dialyzed against PBS. The purity of the preparations was assessed by SDS-PAGE followed by silver staining; sometimes, when albumin contamination was present in the purified preparation, an additional step was added using an Albumin Removal Kit (Pierce). Antibody concentration matching was performed using a densitometric analysis of a silver stain of the purified material. When anti-peptide antibodies obtained from different patients were pooled together and used in functional experiments, each purified patient sample was checked for the presence of the relevant peptide reactivity individually before pooling.

### Affinity Purification of Anti-Transglutaminase Antibodies

Commercially available recombinant human tTG (GST-transglutaminase II fusion protein from *E. coli,* Labvision, Fremont, California, United States) was coupled to Sepharose 4B (Pharmacia), 4 mg protein per gram of dried Sepharose powder, according to manufacturer's instructions. Serum Igs diluted in PBS were applied to the columns. The columns were washed with PBS. Bound Ig were eluted with 0.1 M glycine (pH 2.5) and dialyzed against PBS. For this set of experiments, two groups of serum Igs were used; the first one included five patients' sera that bound tTG but did not recognize the celiac peptide in ELISA. Pooled serum Igs were used to purify anti-tTG antibodies. The antibody preparation obtained was checked in ELISA to assess its negativity on a plate coated with the celiac peptide. The antibodies did not bind the celiac peptide; however, they retained anti-tTG activity assessed using the commercially available Eurospital kit (Trieste, Italy). The antibody preparation contained mostly IgA and approximately 10% of IgG specific for tTG. The second group of sera contained antibodies specific for tTG but failed to recognize both recombinant desmoglein 1 and the desmoglein-derived peptide (LSSLGGTASIGH). Pooled sera from five such patients was used to purify anti-tTG antibodies. The antibody preparation did not bind desmoglein and the desmoglein 1 peptide; however, it still recognized tTG. In order to remove any residual anti-celiac/desmoglein peptide antibody activity, the purified anti-tTG antibody preparations were then repeatedly applied either to a celiac peptide-coupled Sepharose column or to a desmoglein peptide-coupled Sepharose column until the eluate optical density (OD) reading was less than 0.0025.

### ELISA

The direct and competitive ELISA for antibody binding to the synthetic peptides have already been described elsewhere [23]. Briefly, the synthetic peptides were used at a concentration of 20 μg/ml in PBS to coat polystyrene plates (Nunc, Roskilde, Denmark). Plates were then blocked for 1 h with PBS 3% BSA. Serum samples were diluted in diluting buffer (PBS 1% BSA 0.05% Tween) and incubated on the plates for 3 h at room temperature. Plates were then washed twice with PBS 1% Tween and twice with PBS. Bound antibodies were detected by an alkaline phosphatase-conjugated anti-human IgA antiserum (1:1,000 in diluting buffer) (Sigma, St. Louis, Missouri, United States) or with an alkaline phosphatase-conjugated anti-human IgG antiserum (1:2,000 in diluting buffer) (Sigma). In some experiments, an alkaline phosphatase-conjugated anti-rabbit IgG antiserum (1:2,000 in diluting buffer) (Sigma) was also used. The secondary reagents were incubated overnight at 4 °C; plates were then washed twice with PBS/Tween and twice with PBS. The alkaline phosphatase substrate (Sigma) diluted in bicarbonate buffer was then added to the wells and the plates read after 30 min or 1 h at a spectrophotometer set at 450 nm. For the competitive assay, the amount of antibody (or serum dilution) that gave 50% of the maximum binding to the antigen on the solid phase was pre-incubated for 1 h at 37 °C with different amounts of competitors (20, 10, 5, 2.5, 1.25, 0.6 μg/ml) or buffer (diluting buffer as the one used in the direct ELISA) and then transferred to the antigen-coated plates. The assay was then carried on as the direct binding assay. For the competitive ELISAs presented in this paper in particular, the following experimental conditions were used. (1) Competitive ELISA using tTG-coated plates. Plates coated with human recombinant tTG were purchased from Eurospital. Serum samples in diluting buffer (serum dilution ranged from 1:400 to 1:1,600 corresponding to 50% maximal binding to solid-phase tTG) were pre-incubated with increasing concentration of competitors (synthetic peptides or recombinant human tTG: 20, 10, 5, 2.5, 1.25, 0.6 μg/ml) for 1 h at 37 °C. The mixture was then transferred to the tTG-coated plate. The remainder of the assay was performed as the direct binding assay. Results are expressed as:

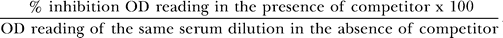
(2) Competitive ELISA with desmoglein 1–coated plates. Desmoglein 1–coated plates were purchased from MBL (Woburn, Massachusetts, United States). Serum samples in diluting buffer (serum dilution ranged from 1:400 to 1:800 corresponding to 50% maximal binding to solid-phase desmoglein 1 were pre-incubated with increasing concentrations of competitors (synthetic peptides and recombinant human tTG: 20, 10, 5, 2.5, 1.25, 0.6 μg/ml) for 1 h at 37 °C. The mixture was then transferred to the desmoglein-coated plate. In this particular assay, we used the desmoglein 1 peptide as liquid-phase competitor, since the only liquid-phase recombinant desmoglein 1 available is an Fc chimera.

In the ELISA for the detection of serum antibodies directed against the peptides, 60 sera diluted 1:200 from normal age- and sex-matched participants were used as the control group. OD values higher than the mean plus three standard deviations (SD) of each serum dilution of the control group were considered positive. Cut-off threshold OD values were 0.200 for anti-celiac peptide IgA antibodies, 0.250 for IgG anti-celiac peptide antibodies, 0.180 for IgA anti-VP-7 peptide, 0.220 for IgG antibodies against VP-7 peptide, 0.210 for IgA antibodies against the desmoglein 1 peptide, 0.260 for IgG antibodies against desmoglein 1 peptide, 0.190 for IgA antibodies against the TLR4 peptide, and 0.220 for IgG antibodies against the TLR4 peptide. Commercially available kits were used to detect anti-tTG antibodies (recombinant human tTG) (Eurospital), (cut-off threshold value for IgA anti-tTG = 7 units/ml), anti-HSP60 antibodies (Stressgen, Victoria, British Columbia, Canada) (cut-off threshold OD value for IgA anti-HSP60 = 0.150), and anti-*Salmonella* antibodies (Bio Quant, San Diego, California, United States) (cut-off threshold OD value for IgG anti-Salmonella typhi = 0.120, and for IgA anti-*Salmonella* = 0.150). Anti-Saccharomyces cerevisiae antibodies (ASCA) have been performed using a commercially available kit (Alpco Diagnostics, Windham, New Hampshire, United States). (Cut-off threshold value for ASCA IgG and IgA is 10 U/ml). Anti-rotavirus antibodies were detected using a commercially available viral extract (Virion, Ruschlikon, Switzerland). The extract was used at a concentration of 25 μg/ml in PBS to coat polystyrene plates (Nunc, Roskilde, Denmark). Plates were then blocked for 1 h with PBS 3% BSA. Serum samples were diluted in diluting buffer (PBS 1% BSA 0.05% Tween) and incubated on the plates for 3 h at room temperature. Plates were then washed twice with PBS 1% Tween and twice with PBS. Bound antibodies were detected by an alkaline phosphatase-conjugated monoclonal anti-human IgG antibody (Zymed). Positive and negative control sera were purchased from Virion. The OD readings after 30 min of colour development were 0.130 for the negative control serum and 0.650 for the positive control. The mean + 3 SD of the control group was an OD of 0.200. Therefore, we considered positive only the sera whose OD readings were higher than 0.200. By using this criteria, some sera of patients with CD were considered negative. However, all of these sera had an OD reading greater than the one observed with the negative control serum, between 0.130 and 0.200; therefore, in theory they are all above the levels of the reference negative serum sample and can be considered borderline.

Anti-rotavirus IgA antibodies were detected similarly, the only difference being the secondary antibody used (alkaline phosphatase-conjugated anti-human IgA antiserum, 1:1,000 in diluting buffer, purchased from Sigma). The OD readings after 30 min of colour development were 0.190 for the negative control serum and 0.550 for the positive control. The mean + 3 SD of the control group was an OD of 0.260. Therefore, we considered positive only the sera whose OD readings were higher than 0.260.

### DELFIA

The dissociation-enhanced lanthanide fluorescent immunoassay (DELFIA) assay is a time-resolved fluorescence method that can be used to study antibody binding to solid-phase proteins or peptides. The celiac peptide was used at a concentration of 20 μg/ml in PBS to coat DELFIA plates (Perkin Elmer). Plates were then blocked for 1 h with a blocking reagent (Perkin Elmer). Serum samples were diluted in diluting buffer (Perkin Elmer) and incubated on the plates overnight at 4 °C. Plates were then washed ten times with washing buffer (Perkin Elmer). Bound antibodies were detected with a europium-labelled anti-human IgA antiserum (1:500 in diluting buffer) (Perkin Elmer) or with a europium-labelled anti-human IgG antiserum (1:500 in diluting buffer) (Perkin Elmer). Plates were read on a Victor 3 instrument (Perkin Elmer) and the data analyzed with software supplied with the DELFIA instrument. For the detection of anti-rotavirus antibodies, the viral extract was used at a concentration of 25 μg/ml in PBS to coat DELFIA plates, and the remainder of the assay was performed as already described. In the DELFIA for the detection of serum antibodies directed against either the celiac peptide or the viral extract, 60 sera diluted 1:200 from normal age- and sex-matched participants were used as the control group. OD values higher than the mean + 3 SD of each serum dilution of the control group were considered positive.

### Detection of Anti-Endomysial Antibodies by Indirect Fluorescent Assay

Anti-endomysial antibodies were determined using a commercially available kit (Diamedix, Miami, Florida, United States) following manufacturer's instructions. Briefly, the antigen slides of primate distal esophagus (endomysial section) were incubated with 50 μl (1 μg/μl) of either purified anti-celiac peptide antibodies cross-reacting with the VP-7 rotavirus peptide or purified anti-VP-7 antibodies for 30 min. After washing for 20 min in PBS, 50 μl of the secondary FITC conjugated anti-human IgA or IgG antibodies were applied to the antigen wells for 30 min.

### Rabbit Antiserum Production

Polyclonal antibodies were generated in New Zealand White rabbits by using standard techniques. Immunizations were performed with a peptide corresponding to VP-7 amino acid residues 260 to 271 (VIQVGGSNVLDI) coupled to the carrier protein keyhole limpet hemocyanin (KLH) (Sigma) and with a peptide corresponding to myotubularin-related protein 2 (MTMR2) amino acid residues 135 to 146 (VEKIGGASSRGE) coupled to KLH. The antisera were tested by ELISA on peptide-coated plates, and the anti-peptide fraction was purified by affinity chromatography on peptide-Sepharose columns. The anti-VP-7 peptide antibodies bound the VP-7 peptide, the TLR4 peptide, and the celiac peptide; moreover, they specifically recognized recombinant tTG and desmoglein 1.

### Western Blot

A rotavirus extract (Virion) was used to detect the VP-7 protein in immunoblot. The extract was enriched for the VP-7 protein by affinity chromatography using a VP-7 peptide Sepharose column. The presence of contaminant tTG in the commercial rotavirus extract was excluded by using the monoclonal anti-tTG purchased from Labvision (clone CUB 7402). A cell lysate of 293T cells transfected with the *TLR4* gene (Cayla-Invivogen) was used to detect TLR4 in Western blot using a TLR4-specific monoclonal antibody (clone 76B357.1, IgG2a, Imgenex, San Diego, California, United States). Blots were probed with primary antibodies followed by either peroxidase-linked anti-human Igs antibodies, anti-human IgA antibodies, anti-rabbit IgG antibodies, or anti-mouse IgG antibodies (all purchased from Sigma). Binding to desmoglein 1 was assessed using the human recombinant desmoglein 1 molecule (Fc chimera) (R&D Systems, Minneapolis, Minnesota, United States) in Western blot. Blots were incubated with biotin-labelled, affinity-purified anti-peptide antibodies followed by peroxidase-labelled avidin, or with patients' sera followed by peroxidase-labelled anti-human IgA antibodies.

### Stimulation of Monocytes

Human monocytes were isolated from normal donors and incubated overnight at a density of 1 × 10^6^ cells/ml in RPMI 10% FCS in 24-well plates in the presence of the antibody preparations (20 μg/ml) or lipopolysaccharide (LPS) (1 μg/ml) (Sigma). The medium used and all the antibody preparations were treated with Detoxi-gel endotoxin-removing columns (Pierce). Presence of LPS contaminants in the antibody preparation and buffers as well as in the culture medium was assessed using the HEK-Blue LPS Detection Kit (Invivogen); all the antibody preparation and buffers used throughout the study were devoided of LPS contaminants. Monocytes were then used for fluorescence-activated cell sorting (FACS) analysis.

### Activation of TLR4 by Antibodies

TLR4 signalling leads to translocation of nuclear factor–kappaB (NF-κB). To monitor the induction of TLR4 signalling in response to ligand stimulation, we have used the pNifty reporter plasmid (Invivogen), expressing the secreted embryonic alkaline phosphatase gene *(SEAP)* under the control of a NF-κB-inducible *ELAM 1* composite promoter [28]. This composite *ELAM 1* promoter is truly NF-κB specific and drives the expression of a reporter gene *(SEAP)* that is induced in the presence of NF-κB and repressed in the absence of the transcription factor. Engineered 293T cells stably transfected with *TLR4* and the co-receptors MD2 and CD14 (Invivogen) were co-transfected with the pNifty plasmids and stable transfectants selected in the presence of phleomycin D1 (Zeocin, Invivogen), as selection agent; 293T cells transfected with *TLR9* (Invivogen) and co-transfected with the pNifty plasmid were used as control. Stable transfectants were grown in DMEM high glucose supplemented with 10% FCS, penicillin-streptomycin, and Normocin (Invivogen). Cells were grown to 60%–80% confluence in growth medium and then harvested and re-suspended in HEK-Blue Detection medium (Invivogen). This medium is specifically designed for detection of NF-κB activation since it turns blue in the presence of phosphatase activity. The product of the reaction is cytotoxic and results in the death of the cells. 2.5 × 10^4^ cells/well (in 200 μl of medium) were plated in 96-well plates in the presence of the appropriate stimuli (purified endotoxin-free antibody preparation, final concentration 4, 2, 1, and 0.5 μg/ml). Positive control for TLR4 was LPS (Invivogen), while the stimulatory oligonucleotide with human-specific type B CpGs (oligodeoxynucleotide 2006 sequence 5′- tcg tcg ttt tgt cgt ttt gtc gtt −3′) (Invivogen) was used for TLR9 cells. Cells were incubated for 24 h at 37 °C in 5% CO_2_ and the blue colour assessed by a spectrophotometer set at 620 nm. All the antibody preparations used in this set of experiments were devoided of LPS contaminants (see previous section). Results are expressed as percentage of positive control, where the positive control is the OD value obtained upon stimulation of *TLR4*-transfected cells with 100 ng/ml LPS (maximal concentration used).

### FACS Analysis

Cells were incubated with R-phycoerythrin directly conjugated anti-CD1, -CD83, and -CD86 antibodies for 30 min on ice. For anti-CD40, antibody binding was revealed using R-phycoerythrin-conjugated secondary antibodies. Samples were run on a FACScan flow cytometer (Becton Dickinson, Mountain View, California, United States). All the antibodies used were purchased from Immunotech (Marseille, France).

### Cytokines Measurement

The levels of interleukin-6 (IL-6), IL-12, and tumour necrosis factor alpha (TNF-alpha) released in the supernatant of cultured monocytes after activation with either LPS or affinity purified anti-celiac peptide antibodies or other stimuli, were determined using commercially available ELISA kits (Biosource International, Camarillo, California, United States) following manufacturer's instructions. The anti-TLR4 monoclonal antibody clone HTA125, isotype IgG2b (Imgenex), was used for inhibition experiments.

### [^3^H] Mannitol Flux

Confluent T84 monolayers grown on 4.7 cm^2^ transwell inserts were incubated in HEPES-phosphate-buffered Ringer solution (HPBR, containing 135 mM NaCl, 5 mM KCl, 3.33 mM NaHPO_4_, 1 mM CaCl_2_, 1 mM MgCl_2_, 10 mM glucose, and 5 mM HEPES [pH 7.4]) for 30 min, followed by treatment with basolaterally applied Igs (20 μg/ml) in the presence of [^3^H] mannitol (5 μCi/ml) in the basolateral compartment. Apical solution was sampled every 30 min (to detect the presence of [^3^H] mannitol) and exchanged with fresh HPBR buffer solution for a total of 4 h. The collected apical samples were placed in vials containing scintillation fluid and analyzed for the presence of [^3^H] mannitol by scintillation counter.

### Statistical Analysis

Frequency of anti-infectious agents antibodies in patients and controls was analyzed by using the chi-square test.

Difference in mannitol concentration in the supernatants of treated and untreated cells at different given times was performed by using the non-parametric Mann-Whitney “U” test.

In all cases, *p*-values less than 0.05 were considered significant.

## Results

### Random Peptide Library

We have screened a dodecamer random peptide library [23] with pooled Igs derived from a panel of 22 patients with active CD after a pre-screening with Igs obtained by the same 22 patients on GFD and negative for the presence of anti-tTG IgA antibodies. By this approach, we aimed to identify only those peptides possibly relevant to the pathogenesis of the disease.

**Figure 1:**
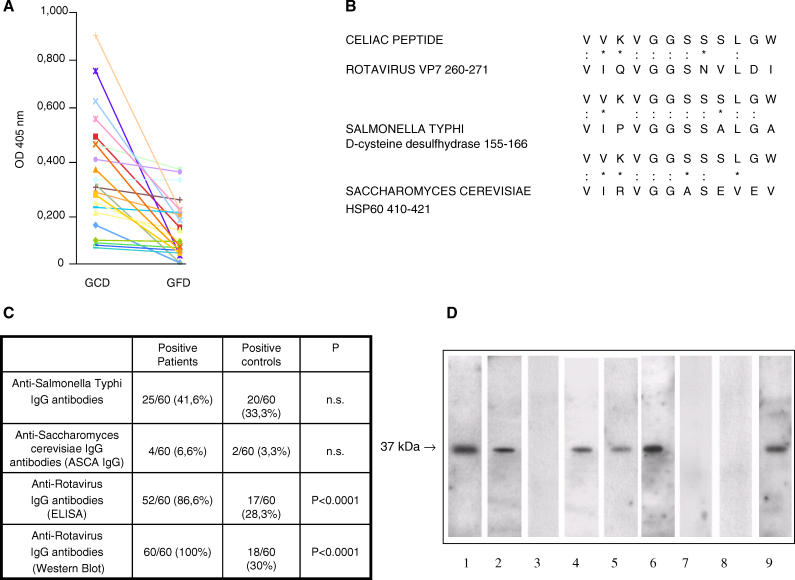
Celiac Peptide Is Recognized by Sera of Patients with Active Disease and Shares Homology with Microbial Antigens (A) The celiac peptide is recognized by serum IgA immunoglobulins of patients on GCD, but not by patients on GFD. Results are expressed as absorbance at 405 nm. (B) Sequence homology between the celiac peptide and infectious agents. The peptide sequence was compared with known microbial protein sequences using the BLASTP via the NCBI BLAST network service (: indicates identity and * indicates conservative substitutions). (C) Frequency of IgG antibodies directed against infectious agents in patients with active CD and in normal healthy controls. (D) Sera of patients with active CD contain IgA antibodies directed against the rotavirus major neutralizing protein VP-7. A rotavirus extract was probed with rabbit antiserum raised against a peptide (VIQVGGSNVLDI) of the VP-7 protein (Lane 1), with affinity-purified anti-celiac peptide antibodies (Lane 2), with antibodies affinity-purified against an irrelevant control peptide (Lane 3), with sera from adult patients with active disease (Lanes 4 and 5), with sera from the same patients on GFD (Lanes 7 and 8), and serum from a 1-y-old child with active CD (Lane 6) and on GFD (Lane 9). A peroxidase-labelled polyvalent anti-human Igs antibody (Lanes 2 and 3) and an anti-human IgA antibody (Lanes 4–9) were used for detection.

We identified a peptide (celiac peptide: VVKGGSSSLGW) that was specifically recognized by serum IgA of 15 out of 22 (68%) individual patients with active disease on GCD and by serum IgA of only four of the same 22 patients on GFD, by both direct and competitive ELISA (Figure 1A). Such reactivity was not detected by the individual sera of 60 healthy individuals. In another group of 38 patients with active disease, whose sera were not used for the screening of the library, 25 individuals (65.7%) had serum IgA against the celiac peptide. The frequency of IgG antibodies against the celiac peptide is 68.3% (41 out of 60 patients). IgA reactivity towards the celiac peptide was not detected in 90 out of 90 patients with systemic sclerosis, and 50 out of 50 patients with systemic lupus erythematosus. We have also tested a panel of sera obtained from patients with small bowel disease: IgA reactivity against the celiac peptide was not detected in 40 out of 40 patients with Crohn disease. IgG antibodies against the celiac peptide were detected in two out of 90 patients with systemic sclerosis, in one out of 50 patients with systemic lupus erythematosus, and in three out of 40 patients with Crohn disease.

These data indicate that this celiac peptide sequence contains an epitope recognized at high frequency by the sera of patients with active disease, but not by patients on GFD.

### Detection of Anti-Celiac Peptide Antibodies by DELFIA

DELFIA is a powerful tool used to analyze antibody binding to a solid-phase antigen; this technique is far more sensitive than ELISA and allows better identification of low–moderate antigen–antibody interactions. We therefore used this method to test the panel of the 22 celiac patients' sera for their ability to recognize the celiac peptide. Using this approach, 20 out of 22 patients' sera had detectable IgA antibodies against the celiac peptide. Out of the five patients' sera that were negative in ELISA, four serum samples showed a moderate positivity towards the celiac peptide, and one patient was only slightly positive, still above threshold. IgA antibodies against the celiac peptide were still detectable in four out of 22 patients with CD in GFD. This reactivity was absent in the 60 controls' sera.

These results indicate that the use of DELFIA in the screening procedure is more sensitive than the use of ELISA and can help to identify sera with moderate antibody binding activity towards a given epitope.

### Anti-Celiac Peptide Antibodies Recognize the Rotavirus-Derived Protein VP-7

Since in CD a transient infection may facilitate the uptake of gluten peptides into the small intestinal mucosa [11], we decided to compare the celiac peptide sequence with known microbial sequences in a protein data bank (Swiss-Prot database) using the BLASTP via the NCBI BLAST network service, and found that the peptide sequence shared a high degree of homology with different proteins, including rotavirus serotype I major neutralizing protein VP-7 [13], S. typhi D-cysteine desulfhydrase, and S. cerevisiae HSP60 (Figure 1B). Two criteria were used in selecting such microbial proteins: the first is the extent of homology calculated by measuring the length of the homologous stretch and the number of matched amino acids (both identities and conservative substitutions were considered); the second one is based on the nature of the infectious agents. We privileged those agents that can be potentially involved in human pathology. Since these infectious agents are all correlated with intestinal damage, we evaluated the prevalence of serum IgG antibodies against such agents in 60 patients with active CD. We found that only four patients had antibodies directed against S. cerevisiae (6.6 %), and 25 patients had antibodies against S. typhi (41,6%), whereas the large majority of patients (52 out of 60: 86.6%) had antibodies against rotavirus in ELISA, and all of them had antibodies against rotavirus in Western blot (Figure 1C). The use of DELFIA partially corrected the discrepancies observed between the ELISA and the Western blot in the detection of IgG anti-rotavirus antibodies. By using DELFIA, 58 out of 60 (96.6%) patients' sera showed the presence of IgG anti-rotavirus antibodies, while there was no significant modification in the number of normal controls positive for the presence of IgG anti-rotavirus (18 out of 60 controls).

IgA antibodies against *Saccharomyces* were not detected in patients' sera, anti-*Salmonella* antibodies were present in eight out of 60 patients (13.3%), and IgA antibodies against rotavirus were present in 41 out of 60 patients (68.3%). The frequency of IgG antibody reactivity against S. typhi and *Saccharomyces* did not differ among patients and control participants. On the contrary, the frequency of antibodies against rotavirus was significantly higher in patients than in control participants (*p* < 0.0001). Moreover, we have tested the patients' sera for their ability to recognize the VP-7 peptide in ELISA; 55 out of 60 patients' sera (91%) had IgG antibodies against the VP-7 peptide, while IgA antibodies against the peptide were present in 45 out of 60 patients' sera (75%). These data indicate that the VP-7 peptide contains a crucial epitope of the anti-rotavirus antibody response in individuals affected by CD.

We therefore decided to focus our attention on the viral protein for the following experiments. We next isolated the antibody component against the celiac peptide from individual sera of ten patients on GCD by affinity chromatography using a peptide Sepharose column. The affinity-purified antibodies specifically recognized the protein VP-7 (Figure 1D) that represents a major neutralization protein of rotaviruses. IgA antibodies against the viral protein VP-7 were present in 17 out of 22 adult patients with active disease and disappear in all the same patients on GFD. Three children within 1 y of age maintained the presence of anti-VP-7 IgA also on GFD (representative examples are shown in Figure 1D). On the contrary, IgG antibodies against the viral protein VP-7 are present in all the patients with inactive disease (unpublished data).

These results indicate that IgA antibodies directed against the celiac peptide recognize the viral protein only in patients with active disease, suggesting a possible etiological link between viral infection and CD.

### Anti-Celiac Peptide Antibodies Recognize Self-Antigens

**Figure 2:**
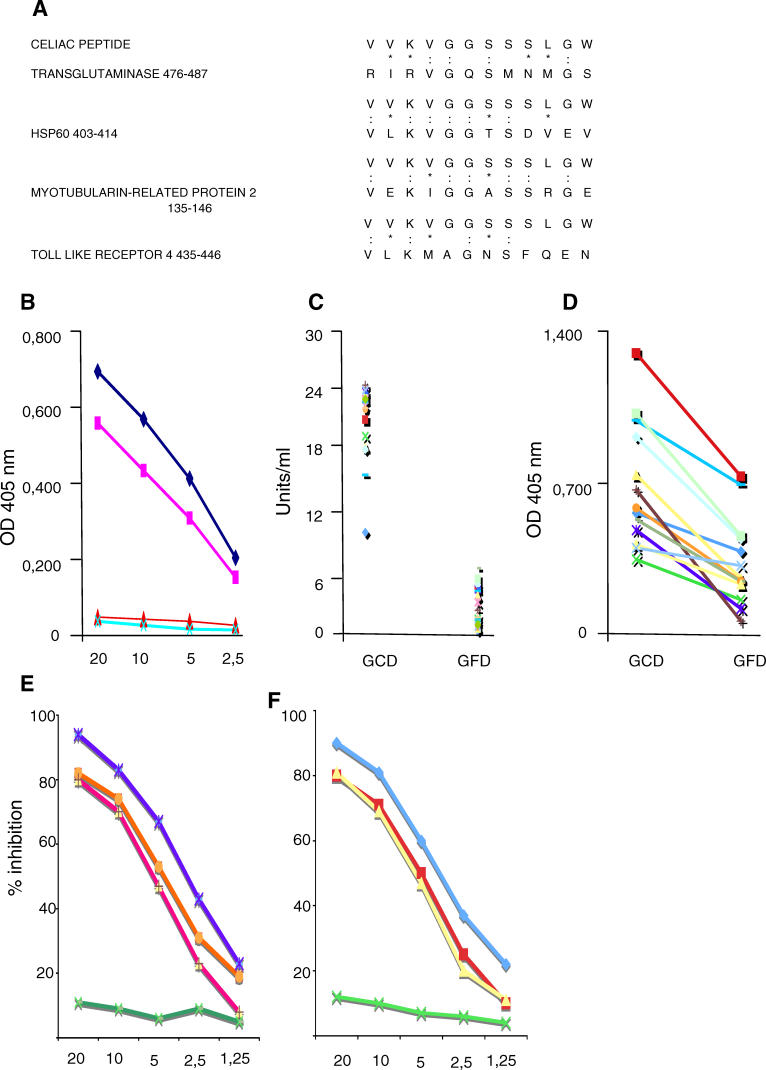
Antibodies against the Celiac Peptide Bind the Self-Antigens tTG and HSP60 (A) Sequence homology between the celiac peptide and self-proteins (colons indicate identity and asterisks indicate conservative substitutions). (B) Direct binding of affinity-purified antibodies against the celiac peptide to recombinant tTG (♦) and to recombinant HSP60 (▪). Binding of affinity-purified antibodies against the control peptide to tTG (▴) and to HSP60 (X). Antibodies against the celiac peptide were purified from ten individual patients with CD; representative example of purified antibodies from one patient is shown. The antibodies obtained from the other nine patients behaved similarly. Data represent absorbance at 405 nm; antibody concentration (horizontal axis), μg/ml. (C) Serum IgA Igs from the 22 patients on GCD, but not from the same patients on GFD, recognize tTG. Data represent U/ml. (D) Serum IgA Igs from the 22 patients on GCD, but not from the same patients on GFD, recognize HSP60. Results are expressed as absorbance at 405 nm. (E) Inhibition of binding of anti-celiac peptide antibodies to solid-phase tTG by liquid-phase tTG peptide (blue line), by celiac peptide (orange line), and VP-7 peptide (red line), but not by an irrelevant control peptide (green line). (F) Inhibition of binding of anti-celiac peptide antibodies to solid-phase HSP60 by liquid-phase tTG peptide (blue line), by celiac peptide (yellow line), and VP-7 peptide (red line), but not by an irrelevant control peptide (green line). The *y*-axis represents percentage of inhibition, and the *x*-axis indicates inhibitor concentration (μg/ml).

As CD is characterized by autoimmune injury in different tissues [2,4], we next compared the celiac peptide sequence with human proteins in a protein data bank (Swiss-Prot database of known human sequences) using the BLASTP via the NCBI BLAST network service, and found that the peptide shared a high degree of homology with different self-antigens, including tTG, HSP60, MTMR2 [29], and TLR4 (Figure 2A).

**Figure 3:**
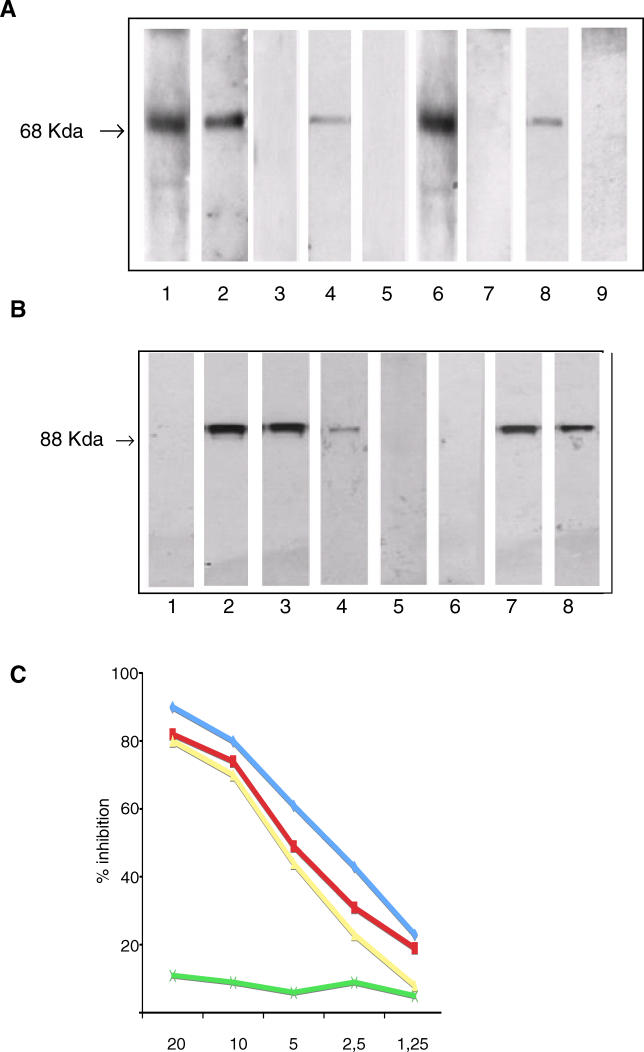
Antibodies against the Celiac Peptide Bind the Self-Antigens MPMR2 and TLR4 (A) Sera of patients with active CD contain IgA antibodies directed against MPMR2; such reactivity is not present in patients on GFD. A K562 cell lysate was probed with rabbit antiserum raised against a peptide (VEKIGGASSRGE) of the MPMR2 (Lane 1), with antibodies affinity-purified against the celiac peptide (lane 2), with antibodies affinity-purified against an irrelevant control peptide (Lane 3), with sera from patients with active disease on GCD (Lanes 4, 6, and 8), and with sera from the same patients on GFD (Lanes 5, 7, and 9). A peroxidase-labelled polyvalent anti-human Igs antibody (Lanes 2 and 3) and an anti-human IgA antibody (Lanes 4–9) were used for detection. (B) Cell lysate from untransfected 293T cells was probed with the monoclonal antibody against TLR4 (Lane 1). Cell lysate from 293T cells transfected with the human *TLR4* gene was probed with a monoclonal antibody directed against TLR4 (Lane 2), and with antibodies affinity-purified against the celiac peptide (Lane 3). Cell lysate from 293T cells transfected with the *TLR4* gene was probed with biotin-labelled anti-TLR4 monoclonal antibody (Lane 4). Cell lysate from 293T cells transfected with the *TLR4* gene was probed first with affinity-purified anti-peptide antibodies, followed by an incubation with biotin-labelled anti-TLR4 monoclonal antibody (Lane 5). Cell lysate from human plasmocytoid dendritic cells was probed with the monoclonal antibody against TLR4 (Lane 6). Cell lysate from human monocytes was probed with the monoclonal antibody against TLR4 (Lane 7) and with affinity-purified anti-peptide antibodies (Lane 8). (C) Inhibition of binding of anti-celiac peptide antibodies to solid-phase TLR4 peptide by liquid-phase TLR4 peptide (blue line), by celiac peptide (red line), and VP-7 peptide (red line), but not by an irrelevant control peptide (green line). The *y*-axis represents percentage of inhibition, and the *x*-axis indicates inhibitor concentration (μg/ml).

Affinity-purified antibodies against the celiac peptide bound the recombinant human tTG and HSP60 (Figure 2B). Moreover, serum IgA antibodies directed against tTG and HSP60 detected in patients with active disease disappear following GFD (Figure 2C and 2D), as already observed for anti-celiac peptide-specific IgA antibodies. The binding of patients' sera Igs to solid-phase tTG and HSP60 was inhibited by pre-incubation of sera with either the tTG peptide, the celiac peptide, or the VP-7 peptide (representative examples are shown in Figure 2E and 2F). IgA antibodies against MTMR2 were present in 14 out of 22 patients with active disease, and such reactivity was lost in 13 out of 14 patients in GFD (Figure 3A). One patient in GFD retained a low reactivity against MTMR2. These findings indicate that the tTG sequence (amino acid 476–487) homologous to the celiac peptide is a key epitope in anti-tTG autoantibody recognition [30]. Indeed, the importance of this area of the molecule in autoantibody recognition has been already described, since deletion of residues 473–496 completely abolished autoantibody reactivity of all the tested celiac sera [31].

The celiac peptide also shares homology with the extracellular region of TLR4, an essential receptor for LPS recognition. Affinity-purified antibodies against the celiac peptide recognized TLR4 both in 293T cells transfected with *TLR4* and in human monocytes (Figure 3B).

Altogether, these results indicate that purified anti-celiac peptide antibodies bind the autoantigen tTG and other self-antigens and that the reactivity of serum IgA to these self-proteins is present only in patients with active disease and disappears on GFD.

### Anti-Celiac and Anti-VP-7 Peptide Antibodies Bind Endomysium

**Figure 4:**
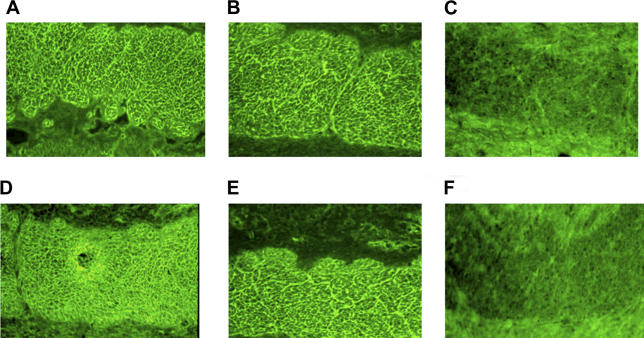
Purified Antibodies Directed against Celiac and VP-7 Peptides Bind Endomysial Structures Pooled affinity-purified antibodies against celiac (A and D) and VP-7 (B and E) peptides from ten patients bind endomysium. Purified antibodies against the irrelevant control peptide from five patients (C and F). (A–C) Slides stained with FITC conjugated anti-human IgA antibodies. (D–F) Slides stained with FITC conjugated anti-human IgG antibodies.

Since the anti-tTG antibodies able to bind endomysial structures may appear early during the onset of the disease, even before the villous atrophy develops, we have tested the ability of purified anti-celiac peptide antibodies and of anti-VP-7 peptide antibodies to bind endomysium. Purified anti-VP-7 antibodies are able to recognize the celiac peptide, tTG, desmoglein, and TLR4; therefore, their possible endomysial binding property would further support the relationship between rotavirus infection and CD. Figure 4A–4F shows that anti-VP-7 peptide antibodies indeed recognize endomysium as well as anti-celiac peptide antibodies.

### Anti-Celiac Peptide Antibodies Induce Monocyte Activation

**Figure 5:**
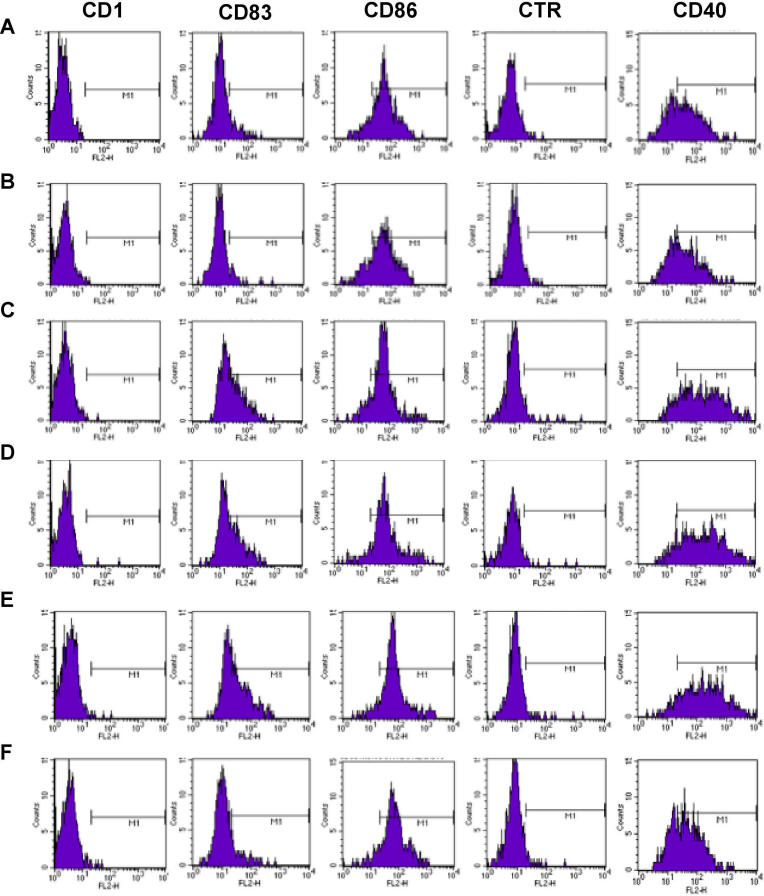
Antibodies against the Celiac Peptide Activate Monocytes FACS analysis of monocytes following incubation with medium alone (A), with antibodies affinity-purified against the control peptide from five patients with CD (B), with LPS (C), antibodies affinity-purified against the celiac peptide from ten patients with active CD (D), pooled Igs from the 22 patients on GCD (E), and pooled Igs from the same patients on GFD (F). Percentage of positive cells = CD83: 9.4% (a), 4.6% (b), 45 % (c), 40% (d), 48% (e), 11.9% (f); CD40: 62.3% (a), 62.3% (b), 89% (c), 88.2% (d), 91.4% (e), 61% (f). Representative example of five independently performed experiments that generated the same FACS profiles. *x*-Axis: FL2-H, fluorescence intensity; *y*-axis: cell counts.

**Figure 6:**
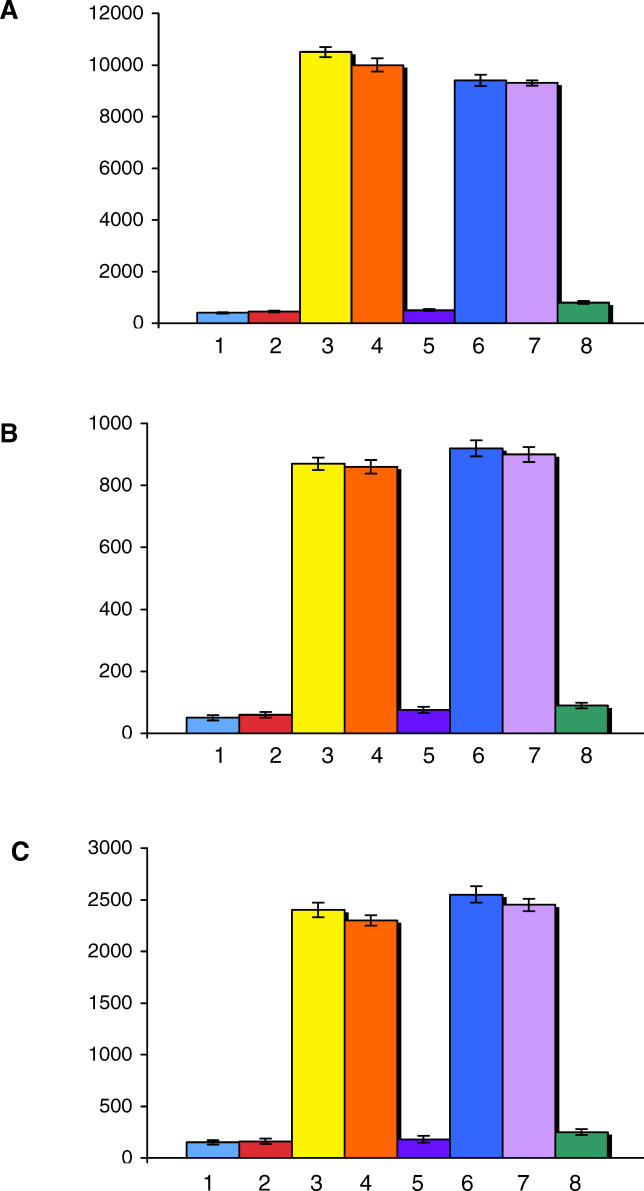
Pro-Inflammatory Cytokines Produced by Activated Monocytes Levels of IL-6 (A), IL-12 (B), and TNF-alpha (C) released in the supernatant by monocytes incubated with medium alone (Line 1), with antibodies directed against an irrelevant peptide (Line 2), with LPS (Line 3), with pooled Igs isolated from the 22 patients with active CD (Line 4), with pooled Igs isolated from the same patients on GFD (Line 5), with purified anti-celiac peptide antibodies obtained from ten patients (line 6), with purified anti-celiac peptide antibodies in the presence of an irrelevant mouse IgG2b antibody (20 μg/ml) (Line 7), and with purified anti-celiac peptide antibodies in the presence of the neutralizing mouse monoclonal antibody anti-TLR4, clone HTA 125 (20 μg/ml) (Line 8). The *y*-axis represents the cytokine concentration expressed as pg/ml. Data represent the mean ±SD of three independently performed experiments.

Since activation of the innate immune system has been suggested to have a significant role in the induction and maintenance of many CD manifestations [8], we next decided to evaluate whether the interaction between affinity-purified anti-celiac peptide antibodies and TLR4 expressed on monocytes could have any functional relevance. Monocytes were therefore incubated overnight either with affinity-purified anti-celiac peptide antibodies, or with antibodies purified against an irrelevant control peptide, or with LPS. The monocyte activation markers considered were evaluated by FACS analysis: in particular, CD83 and CD40 were expressed with a similar fluorescence intensity and by a similar percentage of monocytes after incubation with affinity-purified anti-celiac peptide antibodies, with pooled Igs from patients with active disease and with LPS. These findings were not obtained with pooled Igs from the same patients with inactive disease (Figure 5). These results were confirmed by the measurement of IL-6, IL-12, and TNF-alpha secreted in the medium (Figure 6A–6C). Cytokine production was inhibited by pre-incubation of monocytes with a blocking anti-TLR4 monoclonal antibody, confirming that monocyte activation was indeed mediated by the anti-peptide antibodies through TLR4 engagement.

These data indicate that in CD, autoantibodies are able to bind TLR4 and activate monocytes.

### Anti-Celiac Peptide Antibodies Activate TLR4 in Cells Stably Transfected with the TLR4 Gene

**Figure 7:**
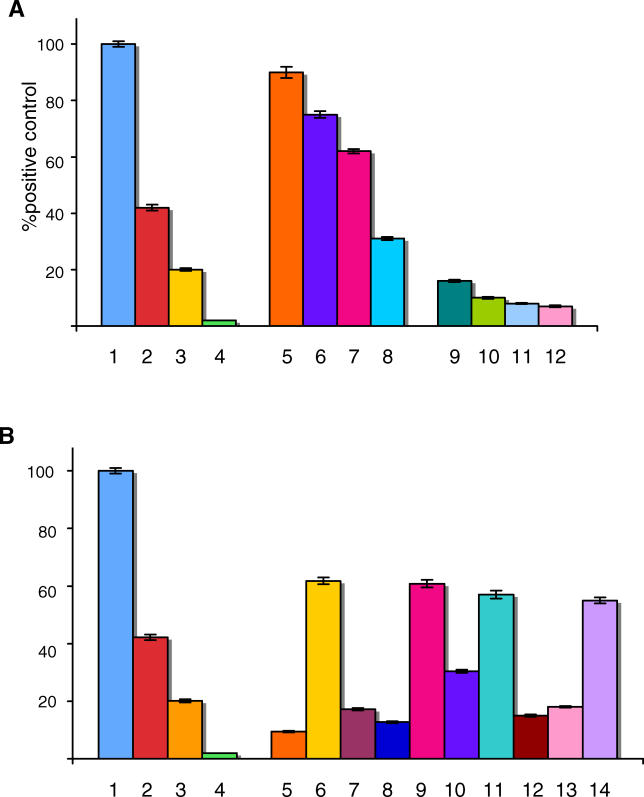
Anti-Celiac Peptide Antibodies Activate TLR4 in Cells Transfected with the *TLR4* Gene Activation of NF-κB upon engagement of TLR4. (A) Stimulation of 293T cells transfected with *TLR4* by LPS 100 ng/ml (1), 10 ng/ml (2), 1 ng/ml (3), 0.1 ng/ml (4); by affinity-purified anti-celiac peptide antibodies, 4 μg/ml (5), 2 μg/ml (6), 1 μg/ml (7), 0.5 μg/ml (8) by affinity-purified antibodies directed against an irrelevant control peptide, 4 μg/ml (9), 2 μg/ml (10), 1 μg/ml (11), and 0.5 μg/ml (12). (B) Stimulation of 293T cells transfected with *TLR4* by LPS 100 ng/ml (1), 10 ng/ml (2), 1 ng/ml (3), 0.1 ng/ml (4), by affinity-purified antibodies against an irrelevant peptide 1 μg/ml (5), by affinity-purified anti-celiac peptide antibodies 1 μg/ml (6), by affinity-purified anti-celiac peptide antibodies 1 μg/ml in the presence of 1 μg/ml anti-TLR4 monoclonal antibody (7), by affinity-purified anti-celiac peptide antibodies 1 μg/ml in the presence of 1 μg/ml celiac peptide (8), by affinity-purified anti-celiac peptide antibodies 1 μg/ml in the presence of 1 μg/ml irrelevant control peptide (9), by affinity-purified anti-celiac peptide antibodies 1 μg/ml in the presence of 1 μg/ml recombinant human tTG (10), by affinity-purified anti-VP-7 peptide antibodies 1 μg/ml (11), by affinity-purified anti-VP-7 peptide antibodies 1 μg/ml in the presence of 1 μg/ml VP-7 peptide (12), by affinity-purified anti-VP-7 peptide antibodies 1 μg/ml in the presence of 1 μg/ml celiac peptide (13), and by affinity-purified anti-celiac peptide antibodies 1 μg/ml in the presence of 1 μg/ml ovalbumin (14). Results are expressed as percentage of positive control, where the positive control is the OD value obtained upon stimulation of *TLR4* transfected cells with 100 ng/ml LPS (maximal concentration used).

293T cells stably transfected with the genes coding for TLR4 and the co-receptors MD2 and CD14 (Invivogen) as well as 293T cells expressing the *TLR9* gene were co-transfected with the pNifty plasmid (Invivogen) and stable transfectants selected with Zeocin. In order to study the ability of antibodies to engage TLRs and activate NF-κB, cells were stimulated in the presence of either specific ligands (LPS for TLR4-positive cells and oligodeoxynucleotides for TLR9-positive cells) or purified-endotoxin-free antibodies. Figure 7 shows the results obtained. Affinity-purified anti-celiac peptide antibodies were able to activate TLR4 in 293T cells transfected with the *TLR4* gene. Similar behaviour was observed for affinity-purified antibodies directed against the VP-7 peptide. On the contrary antibodies affinity purified against the irrelevant peptide and antibodies affinity purified against tTG, which did not recognize the celiac peptide, had no effects on *TLR4-*transfected cells. None of the antibody preparations tested had any effect on *TLR9*-transfected cells (unpublished data). These data show that affinity-purified antibodies against celiac peptide and against the VP-7 peptide have the ability to activate NF-κB upon engagement of TLR4.

### Anti-Celiac Peptide Antibodies Recognize Desmoglein 1

**Figure 8:**
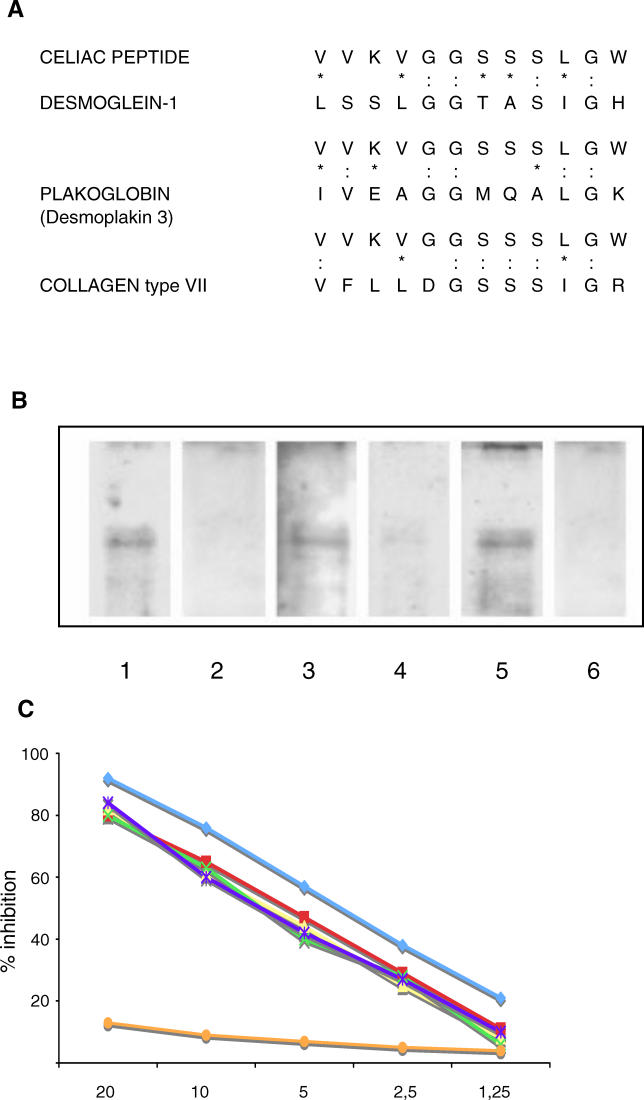
Antibodies against the Celiac Peptide Recognize Desmoglein 1 (A) Sequence homology between the celiac peptide and cell junction proteins. The peptide sequence was compared with known protein sequences using the BLASTP via the NCBI BLAST network service (colons indicate identity and asterisks indicate conservative substitutions). (B) Sera of patients with active CD contain IgA antibodies directed against desmoglein 1; such reactivity is not present in patients on GFD. Recombinant desmoglein 1 was probed with antibodies affinity purified against the celiac peptide (Lane 1), with antibodies affinity purified against an irrelevant control peptide (Lane 2), with sera from patients with active disease on GCD (Lanes 3 and 5), and with sera from the same patients on GFD (Lanes 4 and 6). Biotin-labelled primary antibodies followed by peroxidase-labelled avidin (Lanes 1 and 2) and an anti-human IgA antibody (Lanes 3–6) were used for detection. (C) The binding of affinity-purified anti-celiac peptide antibodies to solid-phase desmoglein 1 is inhibited by desmoglein 1 (blue line), tTG (red line), celiac peptide (purple line), VP-7 peptide (yellow line), and TLR4 peptide (green line), but not by the irrelevant control peptide (orange line). The *y*-axis represents percentage of inhibition, and the *x*-axis indicates inhibitor concentration (μg/ml).

Increased intestinal epithelial cell permeability is a key feature of the early stages of CD, and since the celiac peptide shows sequence homology with cell junction proteins including desmoglein 1 [16], a major component of intercellular desmosome junctions (Figure 8A), we sought to determine whether affinity-purified antibodies against the celiac peptide were able to recognize desmoglein 1. Indeed, the antibodies bound recombinant desmoglein 1 (Figure 8B), and IgA antibodies directed against desmoglein 1 were detected in 18 out of 22 patients' sera. Such reactivity was abolished in all 18 patients' serum samples in GFD. Representative examples of this behaviour are shown in Figure 8B.

In order to investigate whether the anti-celiac peptide antibodies that recognize desmoglein 1 cross-react with tTG and TLR4, a competitive ELISA was performed. The binding of affinity-purified anti-celiac peptide antibodies to solid-phase desmoglein 1 was inhibited by desmoglein 1, tTG, celiac peptide, VP-7 peptide, and TLR4 peptide, but not by the irrelevant control peptide (Figure 8C). These results show that celiac patients' sera contain an antibody population able to recognize desmoglein 1, tTG, TLR4, and rotavirus VP-7 protein.

### Anti-Celiac Peptide Antibodies Induce Increased Epithelial Cell Permeability

**Figure. 9:**
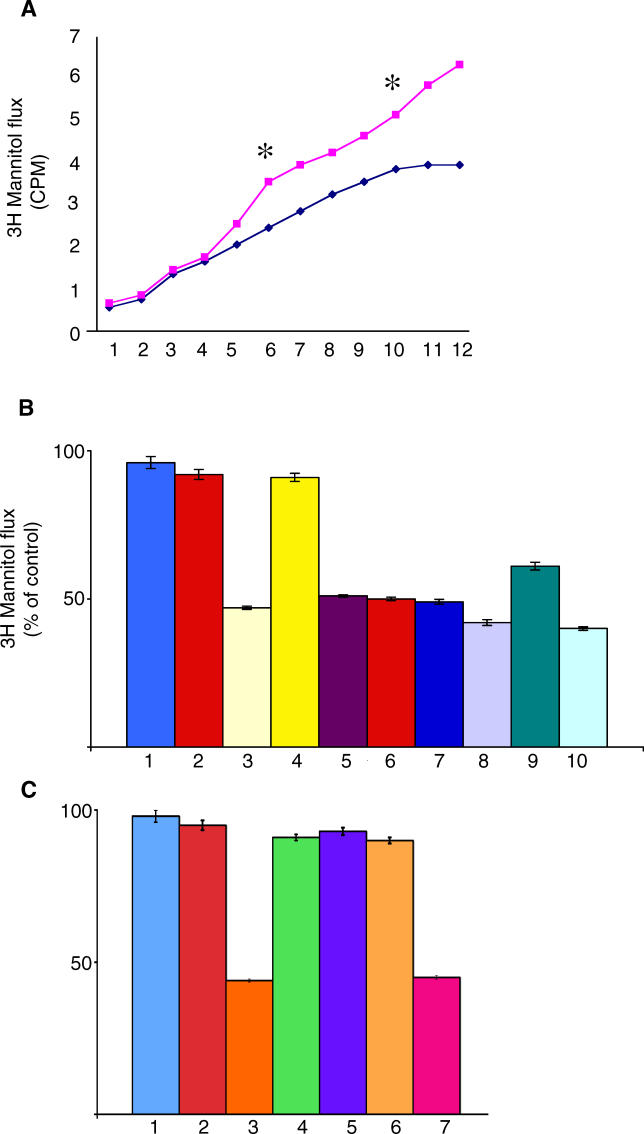
Anti-Celiac Peptide Antibodies Increase Epithelial Cell Permeability Cumulative [^3^H] mannitol flux following treatment with pooled antibodies against the celiac peptide (▪) or against the control peptide (♦) purified from ten patients. Purified antibodies were applied to T84 cells in the presence of [^3^H] mannitol (5 μCi/ml) in the basolateral compartment. Apical buffer was sampled every 30 min over 6 h. * indicates statistically significant difference in mannitol flux at 3 h (*p* = 0.01) and at 5 h (*p* = 0.004). Representative example of three independently performed experiments. (B) Confluent T84 monolayers were treated for 3 h with pooled Igs from healthy individuals (1), affinity-purified antibodies against the control peptide from five patients (2), pooled antibodies affinity purified against the celiac peptide from ten patients (3), pooled antibodies affinity purified against tTG and negative for anti-desmoglein 1 activity from five patients (4), pooled antibodies affinity purified against the desmoglein peptide from five patients (5), pooled antibodies affinity purified against the VP-7 peptide from ten patients (6), pooled antibodies affinity purified against the TLR4 peptide from seven patients (7), pooled Igs from the 22 patients with active CD (8), pooled Igs from the same patients on GFD (9), and TNF-alpha (250 U/ml) (10). (C) Confluent T84 monolayers were treated for 3 h with pooled Igs from healthy individuals (1), affinity-purified antibodies against the control peptide (2), antibodies affinity purified against the celiac peptide (3), and antibodies affinity purified against the celiac peptide in the presence of: human recombinant tTG (4), celiac peptide (5), recombinant desmoglein (6), and an irrelevant peptide (7). Data are mean percentages of control (untreated sample) ±SD. *n* = 6 in duplicates.

We then evaluated whether affinity-purified anti-celiac peptide antibodies were able to alter intestinal epithelial cell permeability. Affinity-purified antibodies against the celiac peptide induced a time-dependent increase in the transepithelial flux of [^3^H] mannitol from the basolateral to the apical compartment (Figure 9A) in T84 human intestinal epithelial cell line [32], used to study the epithelial barrier function. The divergence of flux rate between monolayers treated with antibodies against the celiac peptide and against an irrelevant peptide reached significance after 3 h. This increased permeability was also observed with affinity-purified antibodies directed against the tTG peptide, against desmoglein 1 peptide, against the TLR4 peptide, and against the VP-7 peptide and with pooled Igs from patients with active disease and was similar to the permeability observed after cell incubation with 250 U/ml TNF-alpha. This alteration was present at a much lesser extent with pooled Igs from patients on GFD (Figure 9B). The effect of affinity-purified anti-celiac peptide antibodies on cell permeability was blocked by pre-incubation of antibodies with human recombinant tTG, celiac peptide, and recombinant desmoglein peptide but not by pre-incubation with the irrelevant peptide (Figure 9C).

These data show that in CD there are antibodies able to increase cell permeability upon interaction with junctional proteins such as desmoglein 1.

### Rabbit Antibodies Raised against the VP-7 Peptide Activate TLR4 and Increase Epithelial Cell Permeability

**Figure 10:**
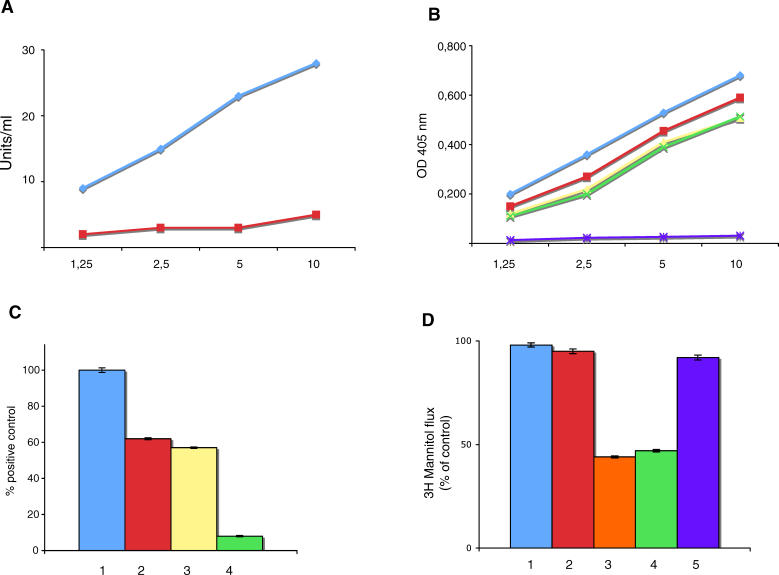
Features of Rabbit Anti-VP-7 Antibodies (A) Binding of rabbit anti-VP-7 antibodies to tTG (blue line). Red line indicates pre-immune rabbit serum. (B) Binding of rabbit anti-VP-7 antibodies to VP-7 peptide (blue line), celiac peptide (red line), desmoglein peptide (yellow line), and TLR4-peptide (green line). Purple line indicates binding to the irrelevant control peptide. (C) TLR4 activation by LPS (100 ng/ml) (1), affinity-purified human anti-celiac peptide antibodies (2), rabbit anti-VP-7 antibodies (3), and pre-immune rabbit serum (4). Results are expressed as percentage of positive control, where the positive control is the OD value obtained upon stimulation of *TLR4* transfected cells with 100 ng/ml LPS (maximal concentration used). (D) Confluent T84 monolayers were treated for 3 h with control normal human Ig (1), affinity-purified human antibodies to the irrelevant control peptide (2), affinity-purified human anti-celiac peptide antibodies (3), rabbit anti-VP-7 antibodies (4), and pre-immune rabbit serum (5).

The rabbit anti-VP-7 peptide antibodies were able to bind human recombinant tTG and celiac, TLR4, and desmoglein 1 peptides (Figure 10A and 10B). Affinity-purified anti-VP-7 peptide antibodies were able to activate TLR4 in 293T cells transfected with the *TLR4* gene (Figure 10C). Moreover, such antibodies were able to increase intestinal epithelial cell permeability (Figure 10D).

These data further support the important role of the rotavirus VP-7 epitope in CD.

## Discussion

Our study aimed to clarify some aspects of the pathogenesis of CD, a small intestinal inflammatory disorder characterized by malabsorption, nutrient deficiency, and a vast array of clinical manifestations. Many of the components and events that generate the altered immune response to gluten have been well characterized; however, there are some features relevant to CD pathogenesis that are still unclear, including the systemic autoimmune aggression, the role played by infectious agents, and the involvement of innate immunity in the different phases of the disease [10]. In our attempt to answer these questions, we used a random peptide library approach that we have already applied to the study of other autoimmune disease conditions for the identification of disease-relevant self-antigens [23–26]. Using this approach, we identified a pathogenetically relevant autoantigen peptide recognized by the sera of patients with active CD. Such peptide shares homology with the rotavirus-derived protein VP-7; human rotaviruses are the most frequent etiologic agents of gastroenteritis in infants and young children in most parts of the world [33]. Anti-celiac peptide antibodies from patients with CD recognize the viral product, suggesting a possible link between rotavirus infection and CD. Moreover, anti-rotavirus IgG antibodies were detected in all patients with CD and in only 32% of control participants, using Western blot. Rotavirus infection has already been associated with pancreatic islet autoimmunity through a mechanism of molecular mimicry [34–36] between viral peptide sequences, in particular within the VP-7 protein and T cell epitopes in the islet cell autoantigen glutamate decarboxylase (GAD) and tyrosine phosphatase IA-2 [37,38]. Moreover, immunization of Lewis rats with a peptide derived from the rotavirus protein VP4, showing homology with the highly pathogenic peptide from retinal S-antigen, induced autoimmune uveitis by triggering cross-reactive T cells [39].

We report here that purified anti-rotavirus VP-7 peptide antibodies are able to cross-react with the celiac peptide, desmoglein peptide, and TLR4 peptide. Moreover, such antibodies bind endomysial structures and have functional properties similar to the anti-celiac peptide antibody preparation because they activate TLR4 and alter cell permeability. Finally, we have also observed that in patients with CD the frequency of IgG and IgA antibodies against the VP-7 peptide is higher than the frequency of antibodies directed against the celiac peptide. Altogether, these data suggest that an anti-rotavirus antibody response is present in celiac patients mainly directed against an epitope within the VP-7 protein. Such response occurs at high frequency when compared with the response towards the celiac peptide, and, therefore, since affinity-purified anti-VP-7 peptide antibodies bind celiac peptide, recognize endomysial structures, activate TLR4, and alter cell permeability, it suggests that the rotavirus VP-7 epitope may be important in determining an anti-virus immune response, able to cross-react with self-antigens and to have functional consequences on TLR4 engagement and intestinal permeability. Therefore, it is likely that a molecular mimicry mechanism may be involved in CD pathogenesis.

The celiac peptide shares homology with tTG, the major autoantigen target in CD; in particular, the homology comprises the amino acid 476–487, an epitope located in the immunodominant C-terminus area of tTG. Indeed, the deletion of amino acid 476–496 has been reported to completely abolish tTG binding by anti-tTG autoantibodies in CD patients [31,40]. Therefore, with our approach we have further restricted the immunodominant epitope to residues 476–487.

The celiac peptide also shares homology with other self-antigens such as HSP60, MTMR2, and TLR4.

Heat-shock proteins are highly conserved proteins synthesized when cells are exposed to stressful stimuli, such as infection and inflammation [15], and increased expression of HSPs has been observed in jejunal epithelial cells in patients with CD [41]. Antibodies against the celiac peptide cross-react with HSP60 and may therefore induce epithelial cell cytotoxicity, thus amplifying the damage of the intestinal mucosa with increased intestinal permeability. This is a key feature of the disease and is also induced by the cross-reactive anti-peptide antibodies.

MTMR2 belongs to the protein-tyrosine phosphatase family. Defects in MPMR2 are the cause of Charcot-Marie-Toot disease type 4, which is an autosomal recessive demyelinating neuropathy [42]. A demyelinating nervous system disease can be observed in patients with CD [20,21].

Finally, TLRs are type I transmembrane proteins involved in innate immunity by recognition of conserved microbial structures. Activation of antigen presenting cells via innate immune receptors such as TLR4 can break self-tolerance and trigger the development of autoimmunity [43,44]. The anti-celiac peptide antibodies bind TLR4 on monocytes and induce both the expression of activation molecules such as CD83 and CD40 and the production of pro-inflammatory cytokines in a extent similar to LPS. The cytokine secretion is consequent to engagement of TLR4 because the pre-incubation of monocytes with an anti-TLR4 monoclonal antibody abolishes such production.

In conclusion, our data indicate that in active CD, autoantibodies against an epitope of tTG bind other self-antigens and the rotavirus-derived protein VP-7, suggesting a viral involvement in the pathogenesis of the disease through a mechanism of molecular mimicry. Moreover, such self-reactive antibodies are pathogenetically relevant for their ability to alter the intestinal barrier integrity and to activate monocytes. Monocyte activation follows the binding of TLR4, providing a previously unknown mechanism for TLR4 stimulation. These results provide evidence for a link between innate and adaptive immune response in the pathogenesis of CD.

## Supporting Information

Alternative Language Abstract S1. Italian Translation of the Abstract Click here for additional data file. (21 KB DOC)

Protocol S1 BLAST Results (183 KB PDF)

## Abbreviations

CD: celiac disease
DELFIA: dissociation-enhanced lanthanide fluorescent immunoassay
ELISA: enzyme-linked immunosorbent assay
FACS: fluorescence-activated cell sorting
GCD: gluten-containing diet
GFD: gluten-free diet
HLA: human leukocyte antigen
HSP60: heat shock protein 60
Ig: immunoglobulin
IL-[number]: interleukin-[number]
LPS: lipopolysaccharide
MTMR2: myotubularin-related protein 2
NF-κB: nuclear factor–kappaB
OD: optical density
SD: standard deviations
TLR4: Toll-like receptor 4
TNF-alpha: tumour necrosis factor alpha
tTG: tissue transglutaminase
