# Supplementary material for: In Celiac Disease, a Subset of Autoantibodies against Transglutaminase Binds Toll-Like Receptor 4 and Induces Activation of Monocytes
Source: PLoS Med. 2006 Sep 19;3(9):e358. doi: 10.1371/journal.pmed.0030358 (PMC1569884; doi:10.1371/journal.pmed.0030358)
Supplement: Supplementary file 1 [file pmed.0030358.sd002.pdf]

NCBI results of BLAST

BLASTP 2.2.13 [Nov-27-2005]

Reference:
Altschul, Stephen F., Thomas L. Madden, Alejandro A. Schäffer,
Jinghui Zhang, Zheng Zhang, Webb Miller, and David J. Lipman
(1997), "Gapped BLAST and PSI-BLAST: a new generation of
protein database search programs", Nucleic Acids Res. 25:3389-3402.

RID: 1141625112-6143-132542853548.BLASTQ4

Database: Non-redundant SwissProt sequences
192,554 sequences; 71,789,263 total letters

If you have any problems or questions with the results of this search
please refer to the **BLAST FAQs**
Taxonomy reports

Query=
Length=12

Sequences producing significant alignments:		Score (Bits)	E Value
sp|Q96PK2|MACF4_HUMAN	Microtubule-actin crosslinking factor 1, i	18.5	243
sp|Q9UBV4|WNT16_HUMAN	Wnt-16 protein precursor	18.1	318
sp|Q14669|TRIPC_HUMAN	Thyroid receptor-interacting protein 12 (T	18.1	318
sp|Q16760|DGKD_HUMAN	Diacylglycerol kinase delta (Diglyceride...	18.1	318
sp|P54296|MYOM2_HUMAN	Myomesin-2 (M-protein) (165 kDa titin-a...	18.1	318
sp|Q13614|MTMR2_HUMAN	Myotubularin-related protein 2	18.1	318
sp|Q9UIQ6|LCAP_HUMAN	Leucyl-cystinyl aminopeptidase (Cystinyl...	17.7	415
sp|P49619|DGKG_HUMAN	Diacylglycerol kinase, gamma (Diglycerid...	17.7	415
sp|Q9Y6T7|DGKB_HUMAN	Diacylglycerol kinase, beta (Diglyceride...	17.7	415
sp|Q969S2|NEIL2_HUMAN	Endonuclease VIII-like 2 (Nei-like 2) (...	17.3	542
sp|Q8WUM9|S20A1_HUMAN	Sodium-dependent phosphate transporter ...	17.3	542
sp|O75912|DGKI_HUMAN	Diacylglycerol kinase, iota (Diglyceride...	17.3	542
sp|P52429|DGKE_HUMAN	Diacylglycerol kinase, epsilon (Diglycer...	17.3	542
sp|P52824|DGKQ_HUMAN	Diacylglycerol kinase, theta (Diglycerid...	17.3	542
sp|Q9NZ20|PA2G3_HUMAN	Group III secretory phospholipase A2 pr...	17.3	542
sp|Q13574|DGKZ_HUMAN	Diacylglycerol kinase, zeta (Diglyceride...	17.3	542
sp|Q9P232|CNTN3_HUMAN	Contactin-3 precursor (Brain-derived im...	17.3	542
sp|Q8IY67|RAVR1_HUMAN	Protein raver-1	16.9	708
sp|Q14653|IRF3_HUMAN	Interferon regulatory factor 3 (IRF-3)	16.9	708
sp|P10809|CH60_HUMAN	60 kDa heat shock protein, mitochondrial...	16.9	708
sp|P23743|DGKA_HUMAN	Diacylglycerol kinase, alpha (Diglycerid...	16.9	708
sp|P46977|STT3_HUMAN	Oligosaccharyl transferase STT3 subunit ...	16.9	708
sp|O12788|TBL3_HUMAN	WD-repeat protein SAZD (Transducin beta-lik	16.9	708
sp|O14924|RGS12_HUMAN	Regulator of G-protein signaling 12 (RGS12	16.9	708
sp|Q8TE59|ATS19_HUMAN	ADAMTS-19 precursor (A disintegrin and ...	16.5	925
sp|P58215|LOXL3_HUMAN	Lysyl oxidase homolog 3 precursor (Lysy...	16.5	925
sp|Q9NQ84|GPC5C_HUMAN	G-protein coupled receptor family C gro...	16.5	925
sp|Q9Y570|PPME1_HUMAN	Protein phosphatase methylesterase 1 (PME-	16.5	925
sp|Q08397|LOXL1_HUMAN	Lysyl oxidase homolog 1 precursor (Lysy...	16.5	925
sp|Q92522|H1X_HUMAN	Histone H1x	16.5	925
sp|Q04756|HGFA_HUMAN	Hepatocyte growth factor activator precu...	16.5	925
sp|P04150|GCR_HUMAN	Glucocorticoid receptor (GR)	16.5	925
sp|Q9UBY8|CLN8_HUMAN	Protein CLN8	16.5	925
sp|Q92747|ARC1A_HUMAN	Actin-related protein 2/3 complex subun...	16.2	1208

sp|P0C0L4|CO4A_HUMAN	Complement C4-A precursor (Acidic comple...	16.2	1208
sp|P14923|PLAK_HUMAN	Junction plakoglobin (Desmoplakin-3) (Desmo	16.2	1208
sp|Q6VVB1|NHLC1_HUMAN	NHL repeat containing protein 1 (Malin)	16.2	1208
sp|Q14576|ELAV3_HUMAN	ELAV-like protein 3 (Hu-antigen C) (HuC...	16.2	1208
sp|Q9UKQ2|ADA28_HUMAN	ADAM 28 precursor (A disintegrin and me...	16.2	1208
sp|Q8TDB6|DTX3L_HUMAN	Deltex 3-like protein (B-lymphoma- and ...	16.2	1208
sp|Q15413|RYR3_HUMAN	Ryanodine receptor 3 (Brain-type ryanodi...	16.2	1208
sp|Q86XX4|FRAS1_HUMAN	Extracellular matrix protein FRAS1 precurs	16.2	1208
sp|Q9Y6I9|TX264_HUMAN	Testis expressed sequence 264 protein p...	16.2	1208
sp|Q9NUD5|ZCHC3_HUMAN	Zinc finger CCHC domain-containing protein	16.2	1208
sp|O75420|PERQ1_HUMAN	PERQ amino acid rich with GYF domain prote	16.2	1208
sp|Q9P296|C5ARL_HUMAN	C5a anaphylatoxin chemotactic receptor ...	16.2	1208
sp|P17174|AATC_HUMAN	Aspartate aminotransferase, cytoplasmic ...	16.2	1208
sp|P35222|CTNB1_HUMAN	Beta-catenin	16.2	1208
sp|Q9UKY4|POMT2_HUMAN	Protein O-mannosyl-transferase 2 (Dolic...	16.2	1208
sp|Q8NDV7|TNR6A_HUMAN	Trinucleotide repeat-containing gene 6A...	16.2	1208
sp|P0C0L5|CO4B_HUMAN	Complement C4-B precursor (Basic complem...	16.2	1208
sp|Q92545|RW1_HUMAN	RW1 protein	16.2	1208
sp|O60716|CTND1_HUMAN	Catenin delta-1 (p120 catenin) (p120(ct...	16.2	1208
sp|Q9NRD9|DUOX1_HUMAN	Dual oxidase 1 precursor (NADPH thyroid...	15.8	1578
sp|O95136|EDG5_HUMAN	Sphingosine 1-phosphate receptor Edg-5 (...	15.8	1578
sp|O95932|TGM3L_HUMAN	Protein-glutamine gamma-glutamyltransfe...	15.8	1578
sp|P09237|MMP7_HUMAN	Matrilysin precursor (Pump-1 protease) (...	15.8	1578
sp|P22681|CBL_HUMAN	CBL E3 ubiquitin protein ligase (Signal t...	15.8	1578
sp|P12111|CO6A3_HUMAN	Collagen alpha-3(VI) chain precursor	15.8	1578
sp|Q12772|SRBP2_HUMAN	Sterol regulatory element-binding prote...	15.8	1578
sp|P15976|GATA1_HUMAN	Erythroid transcription factor (GATA-bi...	15.8	1578
sp|Q6UVK1|CSPG4_HUMAN	Chondroitin sulfate proteoglycan 4 prec...	15.8	1578
sp|P59046|NAL12_HUMAN	NACHT-, LRR- and PYD-containing protein...	15.8	1578
sp|P02788|TRFL_HUMAN	Lactotransferrin precursor (Lactoferrin)...	15.8	1578
sp|O15067|PUR4_HUMAN	Phosphoribosylformylglycinamidine syntha...	15.8	1578
sp|P12259|FA5_HUMAN	Coagulation factor V precursor (Activated...	15.8	1578
sp|O15013|ARHGA_HUMAN	Rho guanine nucleotide exchange factor 10	15.8	1578
sp|O75122|CLAP2_HUMAN	CLIP-associating protein 2 (Cytoplasmic...	15.8	1578
sp|P17658|KCNA6_HUMAN	Potassium voltage-gated channel subfami...	15.4	2060
sp|P29322|EPHA8_HUMAN	Ephrin type-A receptor 8 precursor (Tyr...	15.4	2060
sp|Q7Z401|MYCPP_HUMAN	C-myc promoter-binding protein	15.4	2060
sp|Q8NHY2|RFWD2_HUMAN	Ring finger and WD repeat domain protei...	15.4	2060
sp|Q9Y4C8|RBM19_HUMAN	Probable RNA-binding protein 19 (RNA-bindi	15.4	2060
sp|Q96PE3|INP4A_HUMAN	Type I inositol-3,4-bisphosphate 4-phos...	15.4	2060
sp|P31942|HNRH3_HUMAN	Heterogeneous nuclear ribonucleoprotein...	15.4	2060
sp|Q9Y2T3|GUAD_HUMAN	Guanine deaminase (Guanase) (Guanine ami...	15.4	2060
sp|P25440|BRD2_HUMAN	Bromodomain-containing protein 2 (RING3 pro	15.4	2060
sp|Q9NRA2|S17A5_HUMAN	Sialin (Solute carrier family 17 member...	15.4	2060
sp|Q5JSP0|FGD3_HUMAN	FYVE, RhoGEF and PH domain-containing pr...	15.4	2060
sp|Q8N349|OR2LD_HUMAN	Olfactory receptor 2L13	15.4	2060
sp|O43187|IRAK2_HUMAN	Interleukin-1 receptor-associated kinase-l	15.4	2060
sp|P78367|NKX32_HUMAN	Homeobox protein Nkx-3.2 (Bagpipe homeobox	15.4	2060
sp|Q14721|KCNB1_HUMAN	Potassium voltage-gated channel subfami...	15.4	2060
sp|Q6UWN8|ISK6_HUMAN	Serine protease inhibitor Kazal-type 6 prec	15.4	2060
sp|Q9NYQ7|CELR3_HUMAN	Cadherin EGF LAG seven-pass G-type rece...	15.4	2060
sp|Q9ULK4|CRSP3_HUMAN	CRSP complex subunit 3 (Cofactor requir...	15.4	2060
sp|O15143|ARC1B_HUMAN	Actin-related protein 2/3 complex subun...	15.4	2060
sp|Q9BZR6|RTN4R_HUMAN	Reticulon-4 receptor precursor (Nogo re...	15.4	2060
sp|Q9Y2U8|MAN1_HUMAN	Inner nuclear membrane protein Man1 (LEM...	15.4	2060
sp|O14511|NRG2_HUMAN	Pro-neuregulin-2, membrane-bound isoform...	15.4	2060
sp|P01574|IFNB_HUMAN	Interferon beta precursor (IFN-beta) (Fibro	15.4	2060
sp|O75355|ENP3_HUMAN	Ectonucleoside triphosphate diphosphohyd...	15.4	2060
sp|P55084|ECHB_HUMAN	Trifunctional enzyme beta subunit, mitoc...	15.4	2060
sp|P78426|NKX61_HUMAN	Homeobox protein Nkx-6.1	15.4	2060
sp|Q14004|CD2L5_HUMAN	Cell division cycle 2-like protein kina...	15.4	2060
sp|Q8NE62|CHDH_HUMAN	Choline dehydrogenase, mitochondrial precur	15.4	2060
sp|Q92887|MRP2_HUMAN	Canalicular multispecific organic anion ...	15.4	2060
sp|Q03828|EVX2_HUMAN	Homeobox even-skipped homolog protein 2 (EV	15.4	2060
sp|Q8WXH0|SYNE2_HUMAN	Nesprin-2 (Nuclear envelope spectrin re...	15.4	2060

```
sp|Q14154|K0141_HUMAN  Protein KIAA0141                              15.4     2060
```

Alignments

```
>sp|Q96PK2|MACF4_HUMAN Microtubule-actin crosslinking factor 1, isoform 4
Length=5938

 Score = 18.5 bits (36),  Expect =   243
 Identities = 6/11 (54%), Positives = 8/11 (72%), Gaps = 0/11 (0%)

Query  2    VKVGGSSSLGW  12
            VK+ G+ S GW
Sbjct  465  VKISGTFSSGW  475


>sp|Q9UBV4|WNT16_HUMAN Wnt-16 protein precursor
Length=365

 Score = 18.1 bits (35),  Expect =   318
 Identities = 6/8 (75%), Positives = 7/8 (87%), Gaps = 0/8 (0%)

Query  5    GGSSSLGW  12
            GGS+S GW
Sbjct  156  GGSASEGW  163


>sp|Q14669|TRIPC_HUMAN Thyroid receptor-interacting protein 12 (TRIP12)
Length=1992

 Score = 18.1 bits (35),  Expect =   318
 Identities = 6/10 (60%), Positives = 6/10 (60%), Gaps = 0/10 (0%)

Query  3    KVGGSSSLGW  12
            K GGSS   W
Sbjct  253  KTGGSSKFDW  262


>sp|Q16760|DGKD_HUMAN Diacylglycerol kinase delta (Diglyceride kinase delta) (DGK-delta)
(DAG kinase delta) (130 kDa diacylglycerol kinase)
Length=1214

 Score = 18.1 bits (35),  Expect =   318
 Identities = 5/12 (41%), Positives = 8/12 (66%), Gaps = 0/12 (0%)

Query  1    VVKVGGSSSLGW  12
            ++   GG  S+GW
Sbjct  374  ILVCGGDGSVGW  385


>sp|P54296|MYOM2_HUMAN Myomesin-2 (M-protein) (165 kDa titin-associated protein) (165
kDa connectin-associated protein)
Length=1465

 Score = 18.1 bits (35),  Expect =   318
 Identities = 4/11 (36%), Positives = 8/11 (72%), Gaps = 0/11 (0%)

Query  2     VKVGGSSSLGW  12
             +++GGS   + W
Sbjct  1277  MRIGGSEEMAW  1287


 Score = 14.2 bits (25),  Expect =  4590
 Identities = 5/8 (62%), Positives = 6/8 (75%), Gaps = 0/8 (0%)
```

```
Query  5     GGSSSLGW  12
             GGS  LG+
Sbjct  738   GGSPILGY  745
```

>sp|Q13614|MTMR2_HUMAN Myotubularin-related protein 2
Length=643

```
 Score = 18.1 bits (35),  Expect =   318
 Identities = 7/11 (63%), Positives = 9/11 (81%), Gaps = 0/11 (0%)

Query  1     VVKVGGSSSLG  11
             V K+GG+SS G
Sbjct  135   VEKIGGASSRG  145
```

>sp|Q9UIQ6|LCAP_HUMAN Leucyl-cystinyl aminopeptidase (Cystinyl aminopeptidase) (Oxytocinase) (OTase) (Insulin-regulated membrane aminopeptidase) (Insulin-responsive aminopeptidase) (IRAP) (Placental leucine aminopeptidase) (P-LAP)
Length=1025

```
 Score = 17.7 bits (34),  Expect =   415
 Identities = 6/12 (50%), Positives = 7/12 (58%), Gaps = 0/12 (0%)

Query  1     VVKVGGSSSLGW  12
             V KVG  +  GW
Sbjct  863   VFKVGAKTDKGW  874
```

>sp|P49619|DGKG_HUMAN Diacylglycerol kinase, gamma (Diglyceride kinase) (DGK-gamma) (DAG kinase gamma)
Length=791

```
 Score = 17.7 bits (34),  Expect =   415
 Identities = 5/12 (41%), Positives = 8/12 (66%), Gaps = 0/12 (0%)

Query  1     VVKVGGSSSLGW  12
             V+   GG  ++GW
Sbjct  487   VLACGGDGTVGW  498
```

>sp|Q9Y6T7|DGKB_HUMAN Diacylglycerol kinase, beta (Diglyceride kinase) (DGK-beta) (DAG kinase beta) (90 kDa diacylglycerol kinase)
Length=804

```
 Score = 17.7 bits (34),  Expect =   415
 Identities = 5/12 (41%), Positives = 8/12 (66%), Gaps = 0/12 (0%)

Query  1     VVKVGGSSSLGW  12
             V+   GG  ++GW
Sbjct  491   VLACGGDGTVGW  502
```

>sp|Q969S2|NEIL2_HUMAN Endonuclease VIII-like 2 (Nei-like 2) (DNA glycosylase/AP lyase Neil2) (DNA-(apurinic or apyrimidinic site) lyase Neil2) (NEH2)
Length=332

```
 Score = 17.3 bits (33),  Expect =   542
 Identities = 7/8 (87%), Positives = 7/8 (87%), Gaps = 0/8 (0%)

Query  1    VVKVGGSS  8
            VVK GGSS
Sbjct  22   VVKTGGSS  29
```

```
>sp|Q8WUM9|S20A1_HUMAN Sodium-dependent phosphate transporter 1 (Solute carrier family
20 member 1) (Phosphate transporter 1) (PiT-1) (Gibbon ape
leukemia virus receptor 1) (GLVR-1) (Leukemia virus receptor
1 homolog)
Length=679

 Score = 17.3 bits (33),  Expect =   542
 Identities = 6/10 (60%), Positives = 7/10 (70%), Gaps = 0/10 (0%)

Query  3    KVGGSSSLGW  12
            KVG   S+GW
Sbjct  626  KVGSVVSVGW  635


>sp|O75912|DGKI_HUMAN Diacylglycerol kinase, iota (Diglyceride kinase) (DGK-iota) (DAG
kinase iota)
Length=1065

 Score = 17.3 bits (33),  Expect =   542
 Identities = 4/12 (33%), Positives = 8/12 (66%), Gaps = 0/12 (0%)

Query  1    VVKVGGSSSLGW  12
            ++   GG  ++GW
Sbjct  429  ILACGGDGTVGW  440


 Score = 15.4 bits (28),  Expect =  2060
 Identities = 6/7 (85%), Positives = 6/7 (85%), Gaps = 0/7 (0%)

Query  5   GGSSSLG  11
           GGSSS G
Sbjct  67  GGSSSSG  73


>sp|P52429|DGKE_HUMAN Diacylglycerol kinase, epsilon (Diglyceride kinase) (DGK-epsilon)
(DAG kinase epsilon)
Length=567

 Score = 17.3 bits (33),  Expect =   542
 Identities = 5/12 (41%), Positives = 8/12 (66%), Gaps = 0/12 (0%)

Query  1    VVKVGGSSSLGW  12
            V+   GG  ++GW
Sbjct  274  VLVCGGDGTVGW  285


>sp|P52824|DGKQ_HUMAN Diacylglycerol kinase, theta (Diglyceride kinase) (DGK-theta)
(DAG kinase theta)
Length=942

 Score = 17.3 bits (33),  Expect =   542
 Identities = 5/12 (41%), Positives = 8/12 (66%), Gaps = 0/12 (0%)

Query  1    VVKVGGSSSLGW  12
            V+   GG  ++GW
Sbjct  641  VLVCGGDGTVGW  652


>sp|Q9NZ20|PA2G3_HUMAN Group III secretory phospholipase A2 precursor (Phosphatidylcholine
2-acylhydrolase GIII) (GIII sPLA2)
Length=509

 Score = 17.3 bits (33),  Expect =   542
 Identities = 6/11 (54%), Positives = 8/11 (72%), Gaps = 0/11 (0%)
```

```
Query  2    VKVGGSSSLGW  12
            V +GGS +L W
Sbjct  16   VALGGSPALRW  26
```

>sp|Q13574|DGKZ_HUMAN Diacylglycerol kinase, zeta (Diglyceride kinase) (DGK-zeta) (DAG kinase zeta)
Length=1117

```
 Score = 17.3 bits (33),  Expect =   542
 Identities = 4/12 (33%), Positives = 8/12 (66%), Gaps = 0/12 (0%)

Query  1    VVKVGGSSSLGW  12
            ++   GG   ++GW
Sbjct  537  ILACGGDGTVGW  548
```

>sp|Q9P232|CNTN3_HUMAN Contactin-3 precursor (Brain-derived immunoglobulin superfamily protein 1) (BIG-1) (Plasmacytoma-associated neuronal glycoprotein)
Length=1028

```
 Score = 17.3 bits (33),  Expect =   542
 Identities = 7/7 (100%), Positives = 7/7 (100%), Gaps = 0/7 (0%)

Query  3    KVGGSSS  9
            KVGGSSS
Sbjct  553  KVGGSSS  559
```

>sp|Q8IY67|RAVR1_HUMAN Protein raver-1
Length=606

```
 Score = 16.9 bits (32),  Expect =   708
 Identities = 5/11 (45%), Positives = 6/11 (54%), Gaps = 0/11 (0%)

Query  2    VKVGGSSSLGW  12
            V+ GG    GW
Sbjct  536  VRAGGGDMQGW  546
```

>sp|Q14653|IRF3_HUMAN Interferon regulatory factor 3 (IRF-3)
Length=427

```
 Score = 16.9 bits (32),  Expect =   708
 Identities = 6/10 (60%), Positives = 9/10 (90%), Gaps = 0/10 (0%)

Query  1    VVKVGGSSSL  10
            + +VGG+SSL
Sbjct  378  MARVGGASSL  387


 Score = 13.1 bits (22),  Expect = 10225
 Identities = 4/10 (40%), Positives = 6/10 (60%), Gaps = 0/10 (0%)

Query  3    KVGGSSSLGW  12
            +VG  +  GW
Sbjct  232  EVGDRTLPGW  241
```

>sp|P10809|CH60_HUMAN 60 kDa heat shock protein, mitochondrial precursor (Hsp60) (60 kDa chaperonin) (CPN60) (Heat shock protein 60) (HSP-60) (Mitochondrial matrix protein P1) (P60 lymphocyte protein) (HuCHA60)
Length=573

```
 Score = 16.9 bits (32),  Expect =   708
```

```
 Identities = 6/10 (60%), Positives = 9/10 (90%), Gaps = 0/10 (0%)

Query  1    VVKVGGSSSL  10
            V+KVGG+S +
Sbjct  403  VLKVGGTSDV  412
```

>sp|P23743|DGKA_HUMAN Diacylglycerol kinase, alpha (Diglyceride kinase) (DGK-alpha) (DAG kinase alpha) (80 kDa diacylglycerol kinase)
Length=735

```
 Score = 16.9 bits (32),  Expect =   708
 Identities = 4/12 (33%), Positives = 8/12 (66%), Gaps = 0/12 (0%)

Query  1    VVKVGGSSSLGW  12
            ++  GG  ++GW
Sbjct  429  ILVCGGDGTVGW  440
```

>sp|P46977|STT3_HUMAN Oligosaccharyl transferase STT3 subunit homolog (B5) (Integral membrane protein 1) (TMC)
Length=705

```
 Score = 16.9 bits (32),  Expect =   708
 Identities = 5/11 (45%), Positives = 9/11 (81%), Gaps = 0/11 (0%)

Query  1    VVKVGGSSSLG  11
            +V++GGS+  G
Sbjct  599  MVRIGGSTDTG  609
```

>sp|Q12788|TBL3_HUMAN WD-repeat protein SAZD (Transducin beta-like 3 protein)
Length=519

```
 Score = 16.9 bits (32),  Expect =   708
 Identities = 7/11 (63%), Positives = 7/11 (63%), Gaps = 0/11 (0%)

Query  2    VKVGGSSSLGW  12
            V  G SSSL W
Sbjct  408  VASGASSSLPW  418
```

>sp|O14924|RGS12_HUMAN Regulator of G-protein signaling 12 (RGS12)
Length=1447

```
 Score = 16.9 bits (32),  Expect =   708
 Identities = 7/9 (77%), Positives = 8/9 (88%), Gaps = 0/9 (0%)

Query  1    VVKVGGSSS  9
            VV +GGSSS
Sbjct  440  VVDLGGSSS  448
```

>sp|Q8TE59|ATS19_HUMAN ADAMTS-19 precursor (A disintegrin and metalloproteinase with thrombospondin motifs 19) (ADAM-TS 19) (ADAM-TS19)
Length=1207

```
 Score = 16.5 bits (31),  Expect =   925
 Identities = 5/8 (62%), Positives = 6/8 (75%), Gaps = 0/8 (0%)

Query  5   GGSSSLGW  12
           GGS+  GW
Sbjct  55  GGSADPGW  62
```

```
>sp|P58215|LOXL3_HUMAN Lysyl oxidase homolog 3 precursor (Lysyl oxidase-like protein
3)
Length=753

 Score = 16.5 bits (31),  Expect =   925
 Identities = 5/11 (45%), Positives = 7/11 (63%), Gaps = 0/11 (0%)

Query  2    VKVGGSSSLGW  12
            V++GG   L W
Sbjct  432  VQIGGPGPLRW  442


>sp|Q9NQ84|GPC5C_HUMAN G-protein coupled receptor family C group 5 member C precursor
(Retinoic acid-induced gene 3 protein) (RAIG-3)
Length=441

 Score = 16.5 bits (31),  Expect =   925
 Identities = 5/7 (71%), Positives = 6/7 (85%), Gaps = 0/7 (0%)

Query  6    GSSSLGW  12
            G+SS GW
Sbjct  190  GNSSAGW  196


>sp|Q9Y570|PPME1_HUMAN Protein phosphatase methylesterase 1 (PME-1)
Length=386

 Score = 16.5 bits (31),  Expect =   925
 Identities = 5/8 (62%), Positives = 6/8 (75%), Gaps = 0/8 (0%)

Query  5   GGSSSLGW  12
           GG S+L W
Sbjct  85  GGHSALSW  92


>sp|O08397|LOXL1_HUMAN Lysyl oxidase homolog 1 precursor (Lysyl oxidase-like protein
1) (LOL)
Length=574

 Score = 16.5 bits (31),  Expect =   925
 Identities = 5/8 (62%), Positives = 5/8 (62%), Gaps = 0/8 (0%)

Query  5    GGSSSLGW  12
            GG   LGW
Sbjct  302  GGDPRLGW  309


>sp|Q92522|H1X_HUMAN Histone H1x
Length=213

 Score = 16.5 bits (31),  Expect =   925
 Identities = 6/10 (60%), Positives = 8/10 (80%), Gaps = 0/10 (0%)

Query  1   VVKVGGSSSL  10
           V K GGS++L
Sbjct  21  VTKAGGSAAL  30


>sp|Q04756|HGFA_HUMAN Hepatocyte growth factor activator precursor (HGF activator)
(HGFA) [Contains: Hepatocyte growth factor activator short chain;
Hepatocyte growth factor activator long chain]
Length=655

 Score = 16.5 bits (31),  Expect =   925
 Identities = 6/7 (85%), Positives = 7/7 (100%), Gaps = 0/7 (0%)
```

```
Query  4    VGGSSSL  10
            +GGSSSL
Sbjct  409  IGGSSSL  415
```

>sp|P04150|GCR_HUMAN Glucocorticoid receptor (GR)
Length=777

```
 Score = 16.5 bits (31),  Expect =   925
 Identities = 6/12 (50%), Positives = 8/12 (66%), Gaps = 0/12 (0%)

Query  1    VVKVGGSSSLGW  12
            +VK  G+SS  W
Sbjct  701  IVKREGNSSQNW  712
```

>sp|Q9UBY8|CLN8_HUMAN Protein CLN8
Length=286

```
 Score = 16.5 bits (31),  Expect =   925
 Identities = 6/12 (50%), Positives = 8/12 (66%), Gaps = 0/12 (0%)

Query  1    VVKVGGSSSLGW  12
            ++K G S SL W
Sbjct  179  LLKAGWSESLFW  190
```

>sp|O92747|ARC1A_HUMAN Actin-related protein 2/3 complex subunit 1A (SOP2-like protein)
Length=370

```
 Score = 16.2 bits (30),  Expect =  1208
 Identities = 5/8 (62%), Positives = 6/8 (75%), Gaps = 0/8 (0%)

Query  5    GGSSSLGW  12
            GGS + GW
Sbjct  199  GGSGTGGW  206
```

>sp|P0C0L4|CO4A_HUMAN Complement C4-A precursor (Acidic complement C4) [Contains: Complement C4 beta chain; Complement C4-A alpha chain; C4a anaphylatoxin; C4b-A; C4d-A; Complement C4 gamma chain]
Length=1744

```
 Score = 16.2 bits (30),  Expect =  1208
 Identities = 6/7 (85%), Positives = 7/7 (100%), Gaps = 0/7 (0%)

Query  2     VKVGGSS  8
             VKVGG+S
Sbjct  1368  VKVGGNS  1374
```

>sp|P14923|PLAK_HUMAN Junction plakoglobin (Desmoplakin-3) (Desmoplakin III)
Length=744

```
 Score = 16.2 bits (30),  Expect =  1208
 Identities = 5/11 (45%), Positives = 8/11 (72%), Gaps = 0/11 (0%)

Query  1    VVKVGGSSSLG  11
            +V+ GG  +LG
Sbjct  347  IVEAGGMQALG  357
```

>sp|Q6VVB1|NHLC1_HUMAN NHL repeat containing protein 1 (Malin)
Length=395

```
 Score = 16.2 bits (30),  Expect =  1208
 Identities = 5/9 (55%), Positives = 6/9 (66%), Gaps = 0/9 (0%)

Query  4    VGGSSSLGW  12
            +GG  SL W
Sbjct  211  IGGQFSLPW  219
```

>sp|Q14576|ELAV3_HUMAN ELAV-like protein 3 (Hu-antigen C) (HuC) (Paraneoplastic cerebellar degeneration-associated antigen) (Paraneoplastic limbic encephalitis antigen 21)
Length=367

```
 Score = 16.2 bits (30),  Expect =  1208
 Identities = 4/8 (50%), Positives = 6/8 (75%), Gaps = 0/8 (0%)

Query  5    GGSSSLGW  12
            GG++  GW
Sbjct  277  GGAAGAGW  284
```

>sp|Q9UKQ2|ADA28_HUMAN ADAM 28 precursor (A disintegrin and metalloproteinase domain 28) (Metalloproteinase-like, disintegrin-like, and cysteine-rich protein-L) (MDC-L) (eMDC II) (ADAM23)
Length=775

```
 Score = 16.2 bits (30),  Expect =  1208
 Identities = 5/8 (62%), Positives = 6/8 (75%), Gaps = 0/8 (0%)

Query  5    GGSSSLGW  12
            GGS +L W
Sbjct  568  GGSDNLPW  575
```

>sp|Q8TDB6|DTX3L_HUMAN Deltex 3-like protein (B-lymphoma- and BAL-associated protein) (Rhysin-2) (Rhysin2)
Length=740

```
 Score = 16.2 bits (30),  Expect =  1208
 Identities = 7/10 (70%), Positives = 8/10 (80%), Gaps = 0/10 (0%)

Query  1    VVKVGGSSSL  10
            V+K GG SSL
Sbjct  506  VLKGGGMSSL  515
```

>sp|Q15413|RYR3_HUMAN Ryanodine receptor 3 (Brain-type ryanodine receptor) (RyR3) (RYR-3) (Brain ryanodine receptor-calcium release channel)
Length=4870

```
 Score = 16.2 bits (30),  Expect =  1208
 Identities = 5/11 (45%), Positives = 6/11 (54%), Gaps = 0/11 (0%)

Query  2     VKVGGSSSLGW  12
             V  GG   +GW
Sbjct  1093  VVTGGDMRVGW  1103
```

>sp|Q86XX4|FRAS1_HUMAN Extracellular matrix protein FRAS1 precursor
Length=4007

```
 Score = 16.2 bits (30),  Expect =  1208
 Identities = 6/10 (60%), Positives = 8/10 (80%), Gaps = 0/10 (0%)

Query  2     VKVGGSSSLG  11
```

```
               V   GGS+S+G
Sbjct  1605  VSPGGSTSVG  1614
```

>sp|Q9Y6I9|TX264_HUMAN Testis expressed sequence 264 protein precursor (Secreted protein ZSIG11)
Length=313

```
 Score = 16.2 bits (30),  Expect =  1208
 Identities = 5/7 (71%), Positives = 6/7 (85%), Gaps = 0/7 (0%)

Query  6    GSSSLGW  12
            G+SS GW
Sbjct  246  GASSRGW  252
```

>sp|Q9NUD5|ZCHC3_HUMAN Zinc finger CCHC domain-containing protein 3
Length=404

```
 Score = 16.2 bits (30),  Expect =  1208
 Identities = 4/8 (50%), Positives = 5/8 (62%), Gaps = 0/8 (0%)

Query  5   GGSSSLGW  12
           GG   +GW
Sbjct  33  GGREKMGW  40
```

>sp|O75420|PERQ1_HUMAN PERQ amino acid rich with GYF domain protein 1
Length=817

```
 Score = 16.2 bits (30),  Expect =  1208
 Identities = 5/8 (62%), Positives = 5/8 (62%), Gaps = 0/8 (0%)

Query  5    GGSSSLGW  12
            GG  S GW
Sbjct  143  GGPRSAGW  150
```

>sp|Q9P296|C5ARL_HUMAN C5a anaphylatoxin chemotactic receptor C5L2 (G-protein coupled receptor 77)
Length=337

```
 Score = 16.2 bits (30),  Expect =  1208
 Identities = 7/9 (77%), Positives = 7/9 (77%), Gaps = 0/9 (0%)

Query  1    VVKVGGSSS  9
            VV  GGSSS
Sbjct  187  VVDYGGSSS  195
```

>sp|P17174|AATC_HUMAN Aspartate aminotransferase, cytoplasmic (Transaminase A) (Glutamate oxaloacetate transaminase 1)
Length=413

```
 Score = 16.2 bits (30),  Expect =  1208
 Identities = 6/9 (66%), Positives = 7/9 (77%), Gaps = 0/9 (0%)

Query  3    KVGGSSSLG  11
            +VGG  SLG
Sbjct  100  RVGGVQSLG  108
```

>sp|P35222|CTNB1_HUMAN Beta-catenin
Length=781

```
 Score = 16.2 bits (30),  Expect =  1208
 Identities = 5/11 (45%), Positives = 8/11 (72%), Gaps = 0/11 (0%)

Query  1    VVKVGGSSSLG  11
            +V+ GG  +LG
Sbjct  357  IVEAGGMQALG  367


>sp|Q9UKY4|POMT2_HUMAN Protein O-mannosyl-transferase 2 (Dolichyl-phosphate-mannose--protein
mannosyltransferase 2)
Length=750

 Score = 16.2 bits (30),  Expect =  1208
 Identities = 5/12 (41%), Positives = 8/12 (66%), Gaps = 0/12 (0%)

Query  1    VVKVGGSSSLGW  12
            +++ GG   LGW
Sbjct  636  LLRGGGQVLLGW  647


>sp|Q8NDV7|TNR6A_HUMAN Trinucleotide repeat-containing gene 6A protein (CAG repeat protein
26) (Glycin-tryptophan protein of 182 kDa) (GW182 autoantigen)
(GW1 protein) (EMSY interactor protein)
Length=1962

 Score = 16.2 bits (30),  Expect =  1208
 Identities = 5/7 (71%), Positives = 6/7 (85%), Gaps = 0/7 (0%)

Query  6     GSSSLGW  12
             GSSS+ W
Sbjct  1602  GSSSVNW  1608




  Database: Non-redundant SwissProt sequences
    Posted date:  Mar 4, 2006  8:15 AM
  Number of letters in database: 7,391,856
  Number of sequences in database:  13,628
Lambda     K      H
   0.310    0.132    0.421
Gapped
Lambda     K      H
   0.267   0.0410    0.140
Matrix: BLOSUM62
Gap Penalties: Existence: 11, Extension: 1
Number of Sequences: 13628
Number of Hits to DB: 59382
Number of extensions: 208
Number of successful extensions: 207
Number of sequences better than 100000: 192
Number of HSP's better than 100000 without gapping: 0
Number of HSP's gapped: 207
Number of HSP's successfully gapped: 207
Length of query: 12
Length of database: 7391856
Length adjustment: 0
Effective length of query: 12
Effective length of database: 7391856
Effective search space: 88702272
Effective search space used: 88702272
T: 11
A: 40
X1: 14 (6.3 bits)
```

```
X2: 38 (14.6 bits)
X3: 64 (24.7 bits)
S1: 14 (10.0 bits)
S2: 14 (10.0 bits)
```
