# Supplementary material for: In Celiac Disease, a Subset of Autoantibodies against Transglutaminase Binds Toll-Like Receptor 4 and Induces Activation of Monocytes
Source: PLoS Med. 2006 Sep 19;3(9):e358. doi: 10.1371/journal.pmed.0030358 (PMC1569884; doi:10.1371/journal.pmed.0030358)
Supplement: Supplementary file 2 [file pmed.0030358.sd001.doc]

**Nella malattia celiaca anticorpi diretti contro la transglutaminasi legano il Toll like receptor 4 e inducono attivazione dei monociti.**

ABSTRACT

**Premesse**

La celiachia e’ una malattia infiammatoria del piccolo intestino che coinvolge diversi organi; e’ sostenuta da una risposta immune al glutine contenuto nella dieta. Gli anticorpi anti-transglutaminasi sono un marker sierologico tipico nei pazienti con malattia in fase attiva e possono scomparire durante il trattamento con dieta priva di glutine. Si e’ avanzata l’ipotesi di un coinvolgimento di agenti infettivi e della immunita’ innata nella patogenesi della malattia, tuttavia tale ipotesi non e’ mai stata confermata. Il mimetismo molecolare e’ uno dei meccanismi con cui agenti infettivi possono indurre una risposta autoimmune.

**Metodi e Risultati**

Nel tentativo di chiarire la patogenesi della malattia celiaca, abbiamo utilizzato una libreria peptidica che esprime peptidi con seuquenza randomizzata; tale libreria e’ stata incubata con un un pool di sieri di pazienti con malattia attiva previa incubazione con i sieri degli stessi pazienti in remissione (in dieta priva di glutine). Con tale procedimento abbiamo identificato un peptide riconosciuto dalle immunoglobuline dei pazienti con malattia attiva, ma non dai pazienti in dieta priva di glutine. Questo peptide presenta omologia di sequenza con la proteina neutralizzante maggiore VP-7 del Rotavirus e con gli autoantigeni transglutaminasi, heat shock protein 60 umana, desmogleina 1 e Toll like receptor 4. Gli anticorpi diretti contro il peptide e purificati per affinita’ dal siero di pazienti con malattia attiva, riconoscono il prodotto virale e gli autoantigeni in ELISA e in Western blot. Questi anticorpi sono in grado di indurre una aumentata permeabilita’ della cellula epiteliale valutata con il flusso transepiteliale di mannitolo marcato con [^3^H] linea cellulare intestinale umana T84. Infine gli anticorpi purificati sono in grado di indurre l’attivazione dei monociti attraverso il legame con il Toll like receptor 4, valutata sia con l’espressione di superficie di marker di attivazione, sia dalla produzione di citokine proinfiammatorie.

**Conclusioni**

Questi risultati dimostrano che nella malattia celiaca in fase attiva un sottopopolazione di anticorpi antitransglutaminasi riconosce la proteina virale VP7 suggerendo un possibile coinvolgimento dell’infezione da Rotavirus nella patogenesi della malattia , attraverso un meccanismo di mimetismo molecolare. Inoltre tali anticorpi riconoscono autoantigeni e sono funzionalmente attivi, essendo in grado di aumentare la permeabilita’ intestinale e di attivare i monociti. Questi dati sono a sostegno del coinvolgimento dell’immunita’ innata nella patogenesi della celiachia attraverso un meccanismo di attivazione del Toll like receptor 4 in precedenza sconosciuto.
